# A Soluble Fucose-Specific Lectin from *Aspergillus fumigatus* Conidia - Structure, Specificity and Possible Role in Fungal Pathogenicity

**DOI:** 10.1371/journal.pone.0083077

**Published:** 2013-12-10

**Authors:** Josef Houser, Jan Komarek, Nikola Kostlanova, Gianluca Cioci, Annabelle Varrot, Sheena C. Kerr, Martina Lahmann, Viviane Balloy, John V. Fahy, Michel Chignard, Anne Imberty, Michaela Wimmerova

**Affiliations:** 1 Central European Institute for Technology, Masaryk University, Brno, Czech Republic; 2 National Centre for Biomolecular Research, Faculty of Science, Masaryk University, Brno, Czech Republic; 3 European Synchrotron Radiation Facility, Grenoble, France; 4 CERMAV-CNRS affiliated to Université de Grenoble, Grenoble, France; 5 Department of Medicine and CVRI, University of California San Francisco, San Francisco, California, United States of America; 6 School of Chemistry, University of Bangor, Bangor, United Kingdom; 7 Unité de Défense Innée et Inflammation, Institut Pasteur and INSERM U874, Paris, France; 8 Department of Biochemistry, Faculty of Science, Masaryk University, Brno, Czech Republic; Drexel University College of Medicine, United States of America

## Abstract

*Aspergillus fumigatus* is an important allergen and opportunistic pathogen. Similarly to many other pathogens, it is able to produce lectins that may be involved in the host-pathogen interaction. We focused on the lectin AFL, which was prepared in recombinant form and characterized. Its binding properties were studied using hemagglutination and glycan array analysis. We determined the specificity of the lectin towards l-fucose and fucosylated oligosaccharides, including α1-6 linked core-fucose, which is an important marker for cancerogenesis. Other biologically relevant saccharides such as sialic acid, d-mannose or d-galactose were not bound. Blood group epitopes of the ABH and Lewis systems were recognized, Le^Y^ being the preferred ligand among others. To provide a correlation between the observed functional characteristics and structural basis, AFL was crystallized in a complex with methyl-α,l-selenofucoside and its structure was solved using the SAD method. Six binding sites, each with different compositions, were identified per monomer and significant differences from the homologous AAL lectin were found. Structure-derived peptides were utilized to prepare anti-AFL polyclonal antibodies, which suggested the presence of AFL on the *Aspergillus*’ conidia, confirming its expression *in vivo*. Stimulation of human bronchial cells by AFL led to IL-8 production in a dose-dependent manner. AFL thus probably contributes to the inflammatory response observed upon the exposure of a patient to *A. fumigatus*. The combination of affinity to human epithelial epitopes, production by conidia and pro-inflammatory activity is remarkable and shows that AFL might be an important virulence factor involved in an early stage of *A. fumigatus* infection.

## Introduction

Fungal infections have accompanied mankind throughout history, but the importance of fungal opportunistic pathogens has increased over the last few decades. With the higher occurrence of immunity-affecting factors, e.g. immunosupressive treatments, AIDS, etc. these widespread yet discrete fungi have become a significant threat. One of these organisms is the saprophytic mold *Aspergillus fumigatus*, responsible for causing allergic diseases, allergic bronchopulmonary aspergillosis or invasive aspergillosis. Together with other *Aspergilli* (*A. flavus*, *A. niger*,…), it attacks immunodeficient humans as well as animal hosts [1,2]. Because of the dissemination of conidiae in the atmosphere, *A. fumigatus* is responsible for airborne infections [3]. Lungs are the most common target, whereas aspergillosis in other organs (skin, heart, kidneys, brain, etc.) is less frequent [4]. Current drugs are based on synthetic fungicides derived from polyenes, azoles and echinocandins [5,6], but the increasing resistance of *A. fumigatus* strains to these treatments means that there is an urgent need for new approaches to be developed.

One of the most abundant groups of proteins involved in the pathogen-host interactions are lectins – proteins of non-immune origin interacting with carbohydrate moieties on the cell surface [7]. Antiadhesive therapy relies on preventing the pathogen binding to the host epithelial cells, consequently leading to the elimination of the pathogen by natural clearance mechanisms. Since this approach does not directly affect the life processes of pathogenic cells, the development of resistance is less probable in this case, which makes lectins very promising drug targets [8].

Carbohydrate-binding proteins from *A. fumigatus* have been investigated and partially characterized in several studies over the last few years [9-13]. The presence of carbohydrate-binding proteins in fungal cultures was clearly demonstrated, whereas a partial description of the lectin binding properties was only given in one case [11]. The recent sequencing of the *A. fumigatus* genome [14,15] opened the way to study adhesion at the molecular level. Based on the sequence similarity with known lectins, it is now possible to perform data mining to identify potential lectins. *Aspergillus fumigatus* lectin (AFL) was identified in the genome of the *A. fumigatus* strain Af293 by a homology search using the *Aleuria aurantia* lectin (AAL) from the “orange-peel” mushroom *Aleuria aurantia* [16]. AAL is a fucose-specific lectin that is very frequently used for the purification or histolabeling of fucosylated glycoconjugates [17]. The crystal structure demonstrated a six-blade β-propeller fold with five fucose binding sites and is the paradigm of a new structural family of lectins [16,18]. Two structural homologues from the opportunistic bacteria *Ralstonia solanacearum* (RSL) [19] and *Burkholderia ambifaria* (BambL) [20] also belong to this lectin family, yet they differ in their β-propeller assembly. *Aspergillus oryzae* lectin (AOL) displays a high sequence similarity to AFL [16] and binds to various fucosylated oligosaccharides [21], however its structure has not yet been solved. AFL was recently identified in native culture of *A. fumigatus* (named as AfuFleA), however, it was not characterized in further detail [22]. AFL is therefore the first characterized lectin in this family of pathogenic fungi, and its study sheds light on the structure-function relationship in fucose-specific β-propellers.

In this paper we describe the production of recombinant AFL. The binding activities were determined by hemagglutination studies and glycan array. Specificity analysis demonstrated binding to a large panel of fucosylated oligosaccharides with a preference for Lewis Y epitopes that are found on human tissues. The 3D structure of AFL has been solved using X-ray diffraction. The subcellular localization of the protein in *A. fumigatus* has been determined, as well as its pro-inflammatory activity on human respiratory epithelial cells.

## Materials and Methods

### Materials

l-Galactose and blood group H II type trisaccharides were purchased from Dextra Laboratories Ltd., other oligosaccharides were purchased from Carbohydrate Synthesis Ltd. Basic chemicals were purchased from Sigma, Duchefa and Applichem companies. Methyl seleno-α,l-fucopyranoside (MeSeFuc) was synthesised as described previously [19]. *A. fumigatus* strain CCM 8338 was obtained from Czech Collection of Microorganisms. Rabbit serum containing primary anti-AFL antibodies was obtained by custom antibody production from Thermo Scientific Pierce (Rockford, IL USA). DyLight488-conjugated goat anti-rabbit IgG was purchased from ImmunoReagents, Inc., Cy3-conjugated goat anti-rabbit IgG from Jackson ImmunoResearch Laboratories, Inc., fucose-polyacrylamide-biotin conjugate from Lectinity and AlexaFluor488-conjugated streptavidin from Life technologies.

### *Aspergilli* genome data analysis

Fungal homologues of AAL lectin from *A. aurantia* were searched in publicly available sequence databases. Both annotated and partially completed genomic data and data from other sources (non-redundant GenBank CDS translations+PDB+SwissProt+PIR+PRF) accessible through NCBI server were searched. Lectin sequence of desire was analyzed according to signal peptide (SignalP 4.0 [23]) and transmembrane region (HMMTOP 2.0 [24]) presence. Sequences of AFL homologues were aligned using ClustalW2.0 [25].

### Expression system construction

Commercially available cDNA library from *A. fumigatus* grown at 37°C (Stratagene) was used as a gene source. The corresponding gene was amplified by PCR with *afl* specific primers (5’-ACCCCGCCATATGTCTACTCCTGGAGCACAG-3’ and 5’-CGAAGCT **TAA**GCAGGAGGAAGAGCACTTCTGC-3’) introducing cleavage sites for NdeI and HindIII restrictases, respectively (underlined) and in-frame stop codon (bold). Amplified product was cloned into the expression vector pET29a (Novagen) using NdeI and HindIII for digestion of both PCR product and vector and T4 ligase for ligation, forming vector pET29-*afl*. *Escherichia coli* cells strain BL21(DE3)Gold (Stratagene) were transformed with the plasmid. The sequence of the plasmid pET29-*afl* and its presence in transformed *E. coli* cells were confirmed by restriction cleavage of re-isolated plasmid and its sequencing.

### Protein expression and purification

*E. coli* BL21(DE3)Gold/pET29-*afl* cells were cultivated in standard low salt LB medium with 50 µg ml^–1^ kanamycin. The overproduction of AFL was induced by addition of isopropyl-β-thiogalactoside to a final concentration of 0.5 mM to the culture grown at 37°C when OD_600_ reached 0.5. After additional three-hour cultivation at 30°C, the cells were harvested using centrifugation (10 min/4°C/6000 g) and pellet was resuspended in 20 mM Tris/HCl, pH 7.3. Cells were desintegrated by sonication and insoluble fractions were removed by centrifugation for 40 min at 4°C at 21,000 g. Protein was purified by affinity chromatography on mannose-agarose column (Sigma-Aldrich). Protein extract was loaded on column equilibrated in 20 mM Tris/HCl, pH 7.3 buffer using FPLC system Akta purifier (GE Healthcare). Protein eluted as a single delayed peak using isocratic conditions and its purity was verified by SDS-PAGE in 15% polyacrylamide gel stained by Coomassie Briliant Blue R-250. Fractions containing pure AFL protein were combined, desalted by dialysis against water and used for further studies or lyophilized for long-term storage.

### Differential scanning microcalorimetry

DSC measurement was performed using VP-DSC (Microcal). Protein sample in MilliQ water (15 µM AFL) with or without ligand was heated at 60°C/hr on the scale from 15°C to 75°C. The reverse scan of the same sample was used to subtract the baseline. Data were evaluated in Origin 7.0 software.

### Analytical ultracentifugation

Sedimentation analysis was performed using ProteomeLab XL-A analytical ultracentrifuge (Beckman Coulter) equipped with An-60 Ti rotor. Before analysis, purified AFL was brought into experimental buffer by dialysis and the dialysate was used as an optical reference.

Sedimentation velocity experiments were performed at various loading concentrations of AFL (0.04 - 0.30 mg·ml^-1^) in 20 mM Tris/HCl, 300 mM NaCl, pH 7.4. Sedimentation velocity experiments were conducted in a standard double-sector centerpiece cell loaded with 420 μl of protein sample and 430 μl of reference solution. Data were collected using absorbance optics at 20° C at a rotor speed of 42,000 rpm. Scans were performed at 280 nm at 6 min interval and 0.003 cm spatial resolution in continuous scan mode. The partial specific volumes of protein together with solvent density and viscosity were calculated from amino acid sequence and buffer composition, respectively, using the software Sednterp (http://bitcwiki.sr.unh.edu). The sedimentation profiles were analyzed with the program Sedfit 13.0 [26]. Continuous size-distribution model for non-interacting discrete species providing a distribution of apparent sedimentation coefficients was used.

Sedimentation equilibrium experiments were performed at 20° C in a six-channel centerpiece cell loaded with 110 µl of AFL (0.05 mg·ml^-1^) and 120 µl of reference solution (20 mM Tris/HCl, 150 mM NaCl, pH 7.4). The sample was gradually spun at rotor speeds of 7,800 rpm, 13,300 rpm, and 23,000 rpm, respectively. After the equilibrium was achieved, data were collected at 280 nm by averaging 20 replicates with 0.001 cm spatial resolution in a step mode. Data from multi-speed experiment were analyzed with SEDPHAT 10.40 [27] using a model for non-interacting discrete species.

### Hemagglutination

Anonymised human blood of blood group A treated with natrium citrate (obtained from Transfusion and Tissue Department, The University Hospital Brno) was washed four times by PBS buffer (137 mM NaCl, 2.7 mM KCl, 8 mM Na_2_HPO_4_, 1.5 mM KH_2_PO_4_, pH 7.4), diluted to 50% by PBS with 0.005% (w/w) natrium azide and stored at 4°C. Prepared red blood cells (RBCs) were used for hemagglutination assay. 2 % (v/v) solution of RBCs in PBS buffer was mixed with the same volume of serially diluted AFL solution to determine the minimal agglutinating concentration. The mixture was incubated for 45 min at room temperature and assessed visually. To determine the minimal inhibitory concentration (MIC) the inhibition assay was carried out as follows: RBCs solution and solution of AFL in concentration of twice minimal hemagglutinating concentration were mixed in PBS buffer in 1:1 ratio and tested saccharides were added to final concentrations of 37.5 - 0.015 mM (l-fucose, methyl-α,l-fucoside, l-galactose, Fuc1-2Gal, Fuc1-3GlcNAc, Fuc1-4GlcNAc, Fuc1-6GlcNAc), 150 - 0.25 mM (d-arabinose, d-fructose, l-arabinose, d-xylose, d-lyxose, d-tagatose, l-rhamnose) and 200 - 0.014 mM (d-mannose, methyl-α,d-mannoside, d-glucose, d-galactose, sialic acid), respectively. Plates were incubated for 45 min at room temperature and analyzed.

### Glycan array

Protein affinity towards biologically important oligosaccharides was determined using glycan array. The pure protein was labelled by fluorescent dye DyLight 488 NHS Ester (Thermo scientific) according to product manual and desalted on Zeba Spin Desalting Column (Thermo scientific). The prepared sample was sent to Consortium for Functional Glycomics (USA). Three different concentrations of AFL (1, 0.2 and 0.05 µg ml^–1^) were analyzed using PA_v4.1 chip. Experimental data were normalized to percentages of the highest RFU value for each analysis and the percentages for each glycan at different lectin concentrations were averaged to obtain an average binding. The data were sorted according to their average binding and analysed.

### Crystallization and data collection

Sitting and hanging drop methods were utilized for screening and optimization of protein crystals, respectively. Freeze-dryed protein was dissolved in water and its activity after resolving was verified by SPR with immobilized Fuc. Various crystallization conditions were tested using crystallization kits from Molecular Dimensions and Qiagen. The final crystals were obtained at 20°C under conditions as follows: 2 µl of precipitant (200 mM CaCl_2_, 25% PEG 4K and 100 mM Tris, pH 8.5) mixed with 1 µl of protein solution containing 15 mg ml^-1^ AFL and 19 mM methyl-α,l-selenofucoside. The crystal growth was initiated by streak-seeding using microcrystals obtained from the same conditions.

### Structure determination

The crystals were cryo-cooled at 100K after soaking them in 15% (v/v) glycerol. Diffraction data were collected at beam-line ID14-4 of the ESRF, Grenoble (France). The data collection and refinement statistics are listed in Table S3. Collected images were processed using XDS [28] and converted to structure factors using the CCP4 programs [29]. The structure of AFL/MeSeFuc was determined using single-wavelength anomalous dispersion at 0.980 Å. Phasing was performed using HKL2Map [30] from the position of 26 Se atoms, corresponding to six selenio-ligands per protein monomer and one selenio-ligand located between two adjacent monomers. After autobuilding cycles using ArpWarp [31], refinement was performed using Refmac [32] alternated with manual model building in Coot [33]. Sugar residues were placed manually and after the addition of water molecules and alternative conformations, the final model has been deposited in the PDB Database with accession number 4agi. 

### Antibody preparation

Based on our AFL structure a set of surface exposed peptides was created with the help of EMBOSS: antigenic program [34]. These peptides were used to immunize rabbits (Thermo Scientific Pierce) and serum with positive anti-AFL peptide titre was collected. The specific Ab’s were affinity purified using peptide-immobilized resin. The activity and specificity of the Ab’s were confirmed using Western blot with recombinant AFL protein as a positive control.

### Immunostaining

Two-weeks *A. fumigatus* culture grown on Czapek-Dox agar at 37°C was used. For immunoblotting, agar plates were washed with 5 g l^-1^ NaCl + 0.002% Tween 20, the suspension was collected and filtered through 30 µm Pierce Centrifuge Column (2 min/1000 g). The flowthrough was centrifuged (2 min/5000 g) and conidia resuspended in binding buffer (20 mM Tris, 100 mM NaCl, 100 µM CaCl_2_, pH 7.7) and desintegrated by 0.1mm glass beads on FastPrep®-24 Instrument (MP Biomedicals). The lysate was mixed with fucose-agarose resin, washed 3x with the binding buffer and strongly interacting proteins were released by washing with sample buffer (50 mM Tris, 2 mM EDTA, 2% SDS, 10% glycerol, 6% mercaptoethanol, 0.2% bromophenol blue, pH 6.8) at 95°C. Samples were analyzed by SDS-PAGE and subsequent blotting onto PVDF membrane treated with rabbit polyclonal anti-AFL serum and ALP-conjugated goat anti-rabbit IgG. Staining was performed in 20 ml reaction buffer (0.1 M Tris, 0.5 mM MgCl_2_, pH 9.5) using mixture of 100 µl 30 mg ml^-1^ BCIP in DMF and 100 µl 60 mg ml^-1^ tetrazolium blue in 70% DMF.

For fluorescence microscopy, two-weeks *A. fumigatus* culture grown on YPG agar at 37°C was used. Freshly collected conidia or hyphae were fixed in 4% formaldehyde in PBS overnight at 4°C, centrifuged (3000 g/5 min) and resuspended in PBS + 0.2% Triton for a 60 minute incubation at RT. Samples were then incubated with anti-AFL or a rabbit IgG isotype control antibody (R&D systems) at 10 µg ml^-1^ in PBS + 1% BSA. Peptide blocking controls were stained as above but also received 50 μg ml^-1^ of an AFL specific peptide used in the antibody production. After 1 hour incubation at RT, sample was washed 3x with PBS and secondary antibody (7 µg ml^-1^ in PBS + 1 % BSA) was added. Two different secondary antibodies were used for different sample batches: DyLight488-conjugated goat anti-rabbit IgG (ImmunoReagents, Inc.) and Cy3-conjugated goat anti-rabbit IgG (Jackson ImmunoResearch Laboratories, Inc.). Sample was incubated at RT for 1 hour, washed 3x with PBS and mounted on slide. Samples were examined using an IX81 motorized inverted microscope (Olympus). The same method was used for the experiment with labeled fucose, where fucose-poylacrylamide-biotin conjugate (Lectinity) was used instead of primary antibodies and AlexaFluor488-conjugated streptavidin (Life technologies) instead of secondary antibodies.

### Evaluation of pro-inflammatory activity

Purified AFL was used for the stimulation of the human bronchial cell line BEAS-2B obtained from the American Type Cell Collection (Manassas, VA). Cells were maintained in serial passage in F-12K culture medium supplemented with 10% FCS, 1% penicillin and streptomycin, 1% glutamine and 10 mM HEPES in 75 cm^2^ culture flasks and seeded at 5x10^4^ on 24-well plates 3 days before stimulation. In all experiments, BEAS-2B cells were stimulated during 15 hours with the different agonists in a 300 ml medium. In sugar-inhibition tests, AFL (0.3 µM) was preincubated for 1 hour with three different concentrations of either l-fucose or d-galactose prior to addition to the cells. IL-8 concentrations in cell culture supernatants were determined using a Duo-Set ELISA kit (R&D Systems, Minneapolis, MN).

## Results

### *Aspergilli* genome data mining

More than 200 *Aspergillus* species have been described so far. Several genome projects have been initiated including *A. fumigatus*, *A. flavus*, *A. niger*, *A. clavatus* and others. Using both annotated and partially completed genomic data and data from other sources (non-redundant GenBank CDS translations+PDB+SwissProt+PIR+PRF), genes coding for a peptide with a strong similarity to the AAL lectin from *A. aurantia* were identified in *A. fumigatus* Af293, A1163, Af10 (identical copies in each strain) and Af210 (two single-point mutations at the protein level compared to three other strains), *A. flavus* NRRL3357 and *A. oryzae* RIB40. Other homologous sequences were identified in related fungal species from the genera *Neosartorya*, *Penicillium*, *Trichophyton*, *Arthroderma*, *Metarhizium* and others (Figure S1). In this study we focused on the protein AFL coded in a commercially available cDNA library from *A. fumigatus* ATCC 13073 (Stratagene). The corresponding gene (*AFUA_5G14740*) from *A. fumigatus* Af293 genome, which was the first to be completed in the genus *Aspergillus*, is located on chromosome 5 and consists of 5 exons. The protein is 314 aa long (without the initial Met) and is predicted not to contain a signal peptide or transmembrane region. Our AFL-coding gene contains three base mutations, that reflects in two amino acid mutations compared to the Af293 derived sequence. Based on alignment with available genome sequences from *A. fumigatus* strains (Figure S2) and repeated cloning, we assumed these mutations to be naturally occurring variations.

### AFL characterization

**Figure 1:**
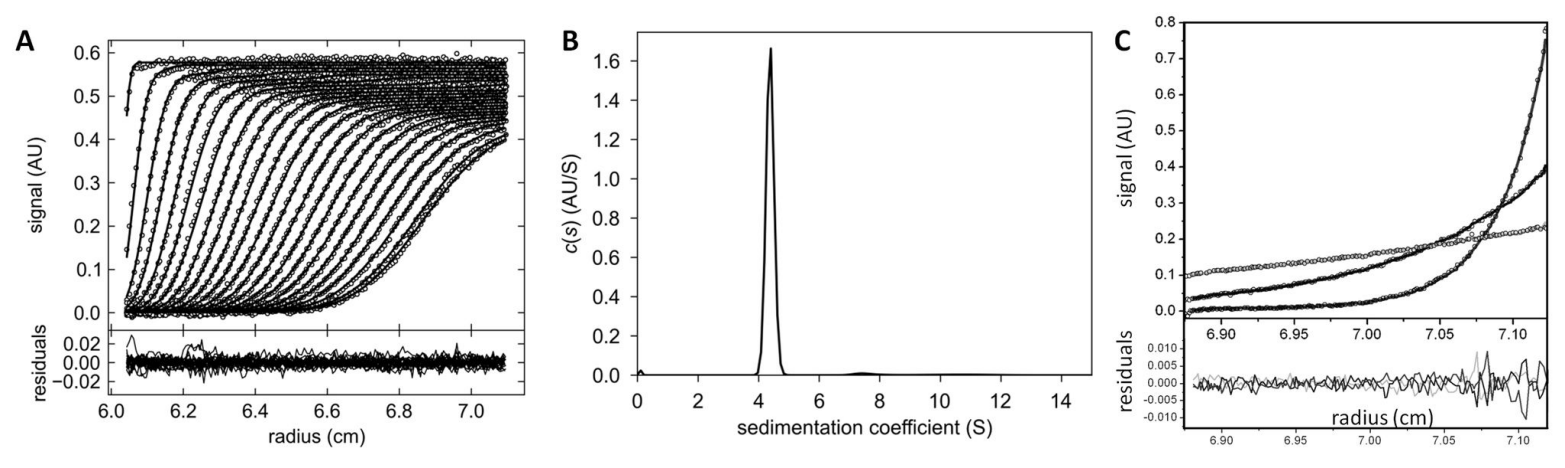
Analytical ultracentrifugation analysis of AFL. (A, B) Sedimentation velocity measurement of AFL. Sedimentation profiles and fitted curves of AFL (0.16 mg·ml^-1^) obtained from continuous c(s) analysis using Sedfit (A, upper panel). Experiment was carried out at 42,000 rpm at 20° C and the scans were recorded every 6 minutes. Every third scan is shown. Residual plot (A, lower panel) shows the differences between experimental data and fitted curves. Continuous size-distribution of sedimenting species (B) provided a value of sedimentation coefficient of 4.38 S for AFL. (s^0^_20,w_ = 4.69 as calculated using Sednterp). The value is clearly much higher than the predicted maximum value for a spherical monomer (3.86 S as calculated in Sednterp) suggesting that a dimer with a moderately elongated shape is formed. The figures were created in GUSSI 1.0.3. (C) Sedimentation equilibrium measurement of AFL. Equilibrium sedimentation distributions of AFL (0.05 mg·ml^-1^) obtained at 20° C at rotor speeds of 7,800 (light curve), 13,300 (dark curve) and 23,000 rpm (grey curve). Residual plot (lower panel) shows the differences between experimental data and fitted curves. Analysis of multi-speed sedimentation equilibrium experiment using SEDPHAT gave a molecular weight of 67.2 kDa, what corresponds to AFL in dimeric form (MW_theor_dimer = 68.9 kDa).

The recombinant AFL protein was prepared using a transformed *E. coli* cell culture. Under the conditions used, the protein was produced mainly in a soluble form. The typical yield reached 10 mg l^–1^ of cell culture. The protein was purified using affinity chromatography on a mannose-agarose column, which enabled isocratic elution and thus avoided contact with fucose or other highly preferred fucosylated moieties. The purity of the protein obtained was confirmed by SDS-PAGE, showing a single band with an apparent size of 34 kDa (Figure S3), which corresponds well with the theoretical weight of 34450 Da. The protein was stable in water at 4°C for more than one week and remained active for more than 6 months once lyophilized and stored at -20°C. The protein is present as a dimer in solution, as determined by sedimentation velocity and equilibrium measurements using analytical centrifugation at various salt concentrations (Figure 1).

The temperature stability was determined using differential scanning calorimetry, which demonstrated a T_m_ of 51°C, while the presence of 4 mM L-fucose (Fuc) increased the T_m_ of the protein to 63°C (data not shown). CD measurements proved AFL to be stable over a wide pH range (4-10) over several days (data not shown).

### Hemagglutination

AFL displayed very strong hemagglutination activity (HU = 17 nM) on human red cells (blood group A). The hemagglutination inhibition assay found AFL to have a strong preference for fucose (Fuc) and methyl-α,l-fucoside (αMeFuc) over the other saccharides tested. The minimal inhibitory concentrations (MIC) observed are listed in Table 1. Fuc and αMeFuc displayed the highest inhibitory effect, followed by l-galactose, d-arabinose and d-fructose. d-mannose is a very weak inhibitor with MIC = 150 mM, whereas other carbohydrates were unable to inhibit hemagglutination at any concentration tested (up to 150 mM). Sialic acid did not inhibit hemagglutination at 18.8 mM, whereas higher concentrations caused hemolysis. Fucosylated disaccharides displayed stronger inhibition potential than free Fuc, with Fuc1-3GlcNAc and Fuc1-4GlcNAc (both with MIC = 15 µM) being slightly better inhibitors than Fuc1-2Gal and Fuc1-6GlcNAc.

**Table 1:** Minimal inhibitory concentrations (MIC) of sugars for AFL.

Sugar	MIC
l-fucose	0.586 mM
Methyl-α,l-fucoside	0.293 mM
Fuc(1,2)Gal (2-O-(l-fucosyl)-d-galactose)	0.293 mM
Fuc(1-3)GlcNAc (3-O-(l-fucosyl)-2-N-acetyl-d-glucosamine)	0.147 mM
Fuc(1-4)GlcNAc (4-O-(l-fucosyl)-2-N-acetyl-d-glucosamine)	0.147 mM
Fuc(1-6)GlcNAc (6-O-(l-fucosyl)-2-N-acetyl-d-glucosamine)	0.293 mM
l-galactose	4.69 mM
d-arabinose	9.375 mM
d-fructose	75 mM
d-mannose	150 mM
methyl-α,d-mannoside, d-glucose, d-galactose, l-arabinose, d-xylose, d-lyxose, d-tagatose, l-rhamnose	NI*

^*^ Carbohydrates did not inhibit hemagglutination at any concentration tested.

### Glycan array

**Figure 2:**
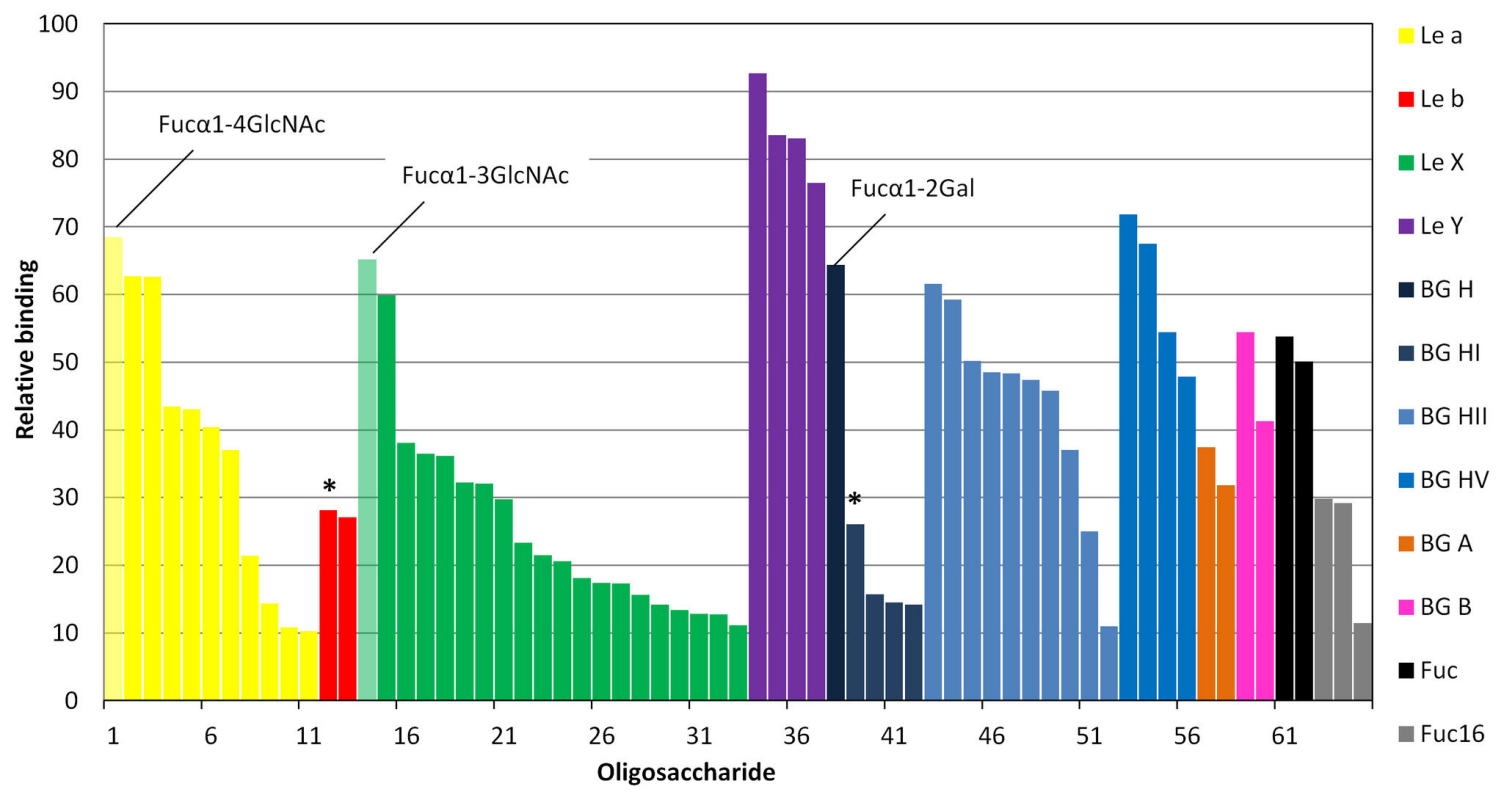
Glycan array results for AFL interaction with biologically important oligosaccharides. The bars show all of an oligosaccharide’s average relative binding above 10 % (see Methods). Immobilized Fuc response given for comparison. BG stands for blood group. Oligosaccharides with mixed epitopes marked with asterisk. Oligosaccharide structures are listed in Table S2 and structures of top binders of each group are shown in Figure S4.

**Figure 3:**
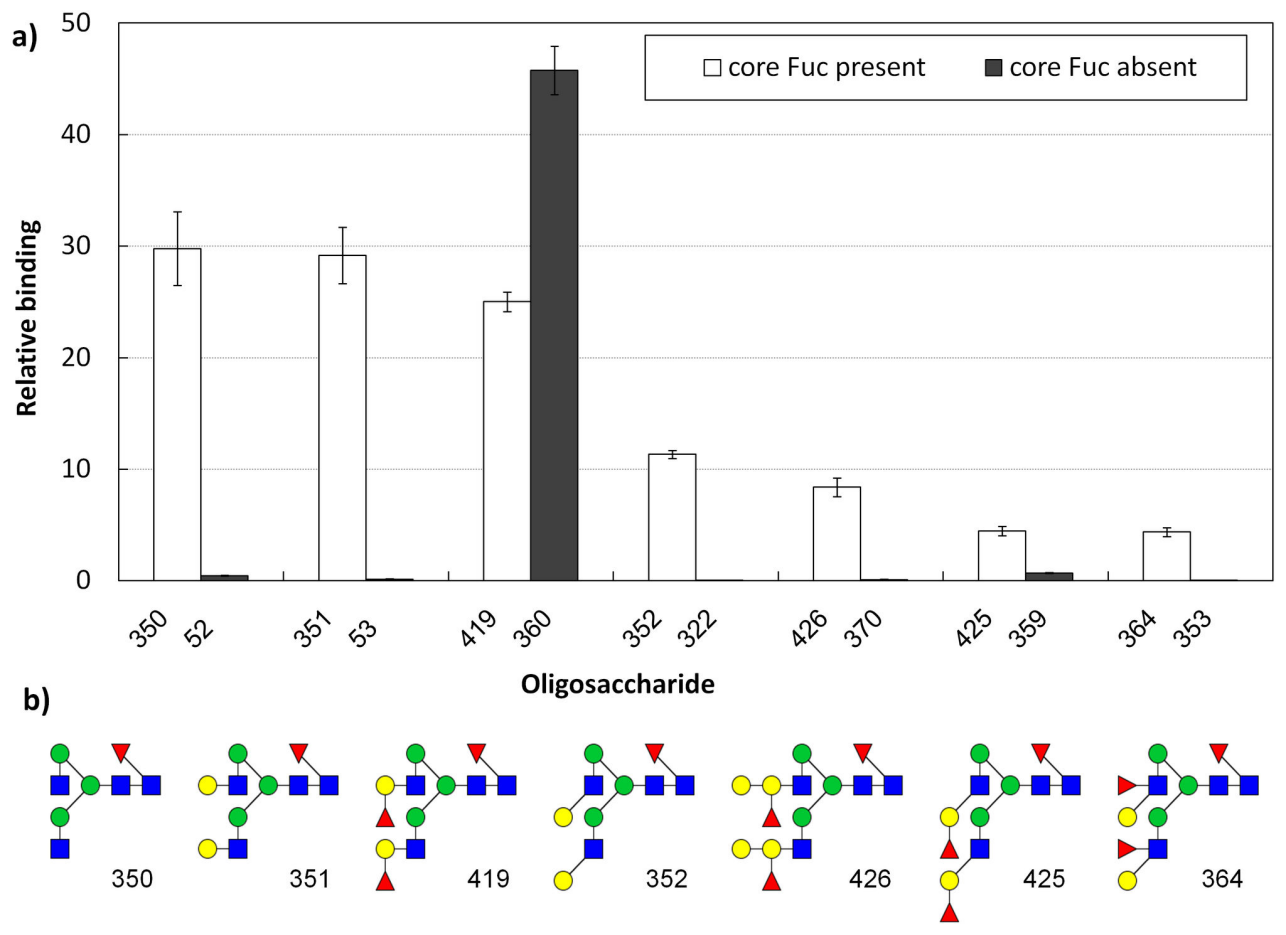
Glycan array analysis of AFL interaction with core α1-6 Fuc. Only saccharides found in both core fucosylated and non-fucosylated forms are included. Corresponding fucosylated forms are shown in schematic representations according to convention by Taylor and Drickamer [44]. The bars show an oligosaccharide’s average relative binding with standard deviation (see Methods). Core α1-6 Fuc saccharides are generally bound better, the exception in the third case is probably caused by the presence of a highly preferred terminal Fuc.

A semi-quantitative measurement was done in collaboration with the Consortium for Functional Glycomics. The binding of AFL (concentrations of 1, 0.2 and 0.05 µg ml^–1^) to the oligosaccharides was tested on a PA_v4.1 chip (Figure 2, 3, S4 and Table S1, S2). AFL bound to all fucose-containing compounds present on the chip, including Fuc and the disaccharides Fucα1-2Gal, Fucα1-3GlcNAc Fucβ1-3GlcNAc and Fucα1-4GlcNAc. Oligosaccharides with terminal Fuc bound through the α1-2, α1-3 and α1-4 linkages were also recognized. The best non-fucosylated binder displayed only 12% binding compared to highest ranked epitopes, whereas all others reach less than 5% relative binding. The only fucosylated glycans that displayed relatively weak binding were those with core α1-6-bound fucose on branched oligosaccharides. However, a comparison of the same oligosaccharide with and without core Fuc revealed significant differences in binding. The non-fucosylated epitope was not recognized, whereas the core α1-6 fucosylated epitope was a weak but significant binding partner (Figure 3). Based on this, we could conclude that α1-6-bound Fuc was also recognized and that the altered response was caused by steric hindrance from part of the oligosaccharide and/or differences in additional protein-oligosaccharide contacts.

Focusing on blood group determinants, AFL binds to all Lewis epitopes (Le^a^, Le^b^, Le^X^, Le^Y^) and blood group A, B and H determinants. Generally, shorter and non-branched oligosaccharides are preferred over larger structures. As for Lewis antigens, immobilized disaccharides Fucα1-3GlcNAc and Fucα1-4GlcNAc were better recognized than any other member of the Le^a^ or Le^X^ group. Only the simplest Le^b^ motif was bound, whereas non-branched Le^Y^ epitopes were the most intensively recognized oligosaccharides of all those tested. In contrast, branched oligosaccharides bearing Le^Y^ epitopes were hardly bound (average rank <3 compared to >75 for non-branched). Simple structures of the ABH blood group system are bound more strongly than complex ones, where a preference was observed for BGH II and BGH V over BGH I. Sialylation and sulfatation generally slightly lower the affinity, but their influence seemed to differ from case to case. Based on the complete data, AFL prefers the Le^Y^ epitope over any of the others tested. Steric hindrance plays an important role in binding, since branched oligosaccharides are bound significantly less than non-branched units, with the exception of Le^Y^, which presents two fucose residues.

### Structure determination

The structure of AFL co-crystallized with methyl-α,l-selenofucoside (MeSeFuc) was determined using single-wavelength anomalous dispersion (SAD) at ESRF, Grenoble, France. The methyl-selenium used for phasing was observed on the bound fucose, as expected, but also in one residue of S-methylselenyl-l-cysteine per monomer, indicating that Cys111 received a seleniomethyl group either from SeMeFuc during soaking, or directly from the solution. The complex crystallized in the P_1_ spacegroup with 4 monomers per unit cell associated in 2 functional units (dimers) and the structure was refined to a resolution of 1.60 Å (Table S3).

### Overall fold

The AFL monomer is composed of 6 blades, each formed of 4 antiparallel β-sheets. Four short α-helixes were found in the structure. They are formed at the N-terminal end (Gly5-Gln8), at the outer edge of blade I (Thr48-Lys50) and blade II (Gly98-Lys103) and in the loop of blade VI (Ser273-Asn277). The overall shape is cylindrical with a 51 Å maximal outer diameter and a height of 40 Å. The central tunnel broadens from 6 Å at the base (N- and C-terminal base) to 12 Å at the other side of the cylinder. The cavity is formed of mainly hydrophobic side chains with the exception of a highly charged base part. The closure of the β-propeller fold occurs mainly via hydrogen bonds formed between the N-terminus and strand 3 of blade I on one side and the middle loop of blade VI and the C-terminus on the other side. This 6-fold β-propeller is structurally similar to that found in the AAL protein [16] with an rmsd of 0.68Å (comparing the A chains of 4agi (AFL) and 1ofz (AAL)). 

Two mutations confirmed by gene sequencing were observed in the 3D structure when compared to the annotated genome of the Af293 strain. R111C is located at the bottom of central tunnel, whereas L20S is situated on the protein surface, more than 8 Å from the closest binding site. As they do not interact with any residues involved in ligand binding or dimerization, they should not affect the binding properties or oligomerization of AFL. Some cysteins are found to be oxidized, since no DTT or similar compound was used during the crystallization. These cysteins, oxidized or not, do not contribute to ligand binding.

### Oligomeric state

**Figure 4:**
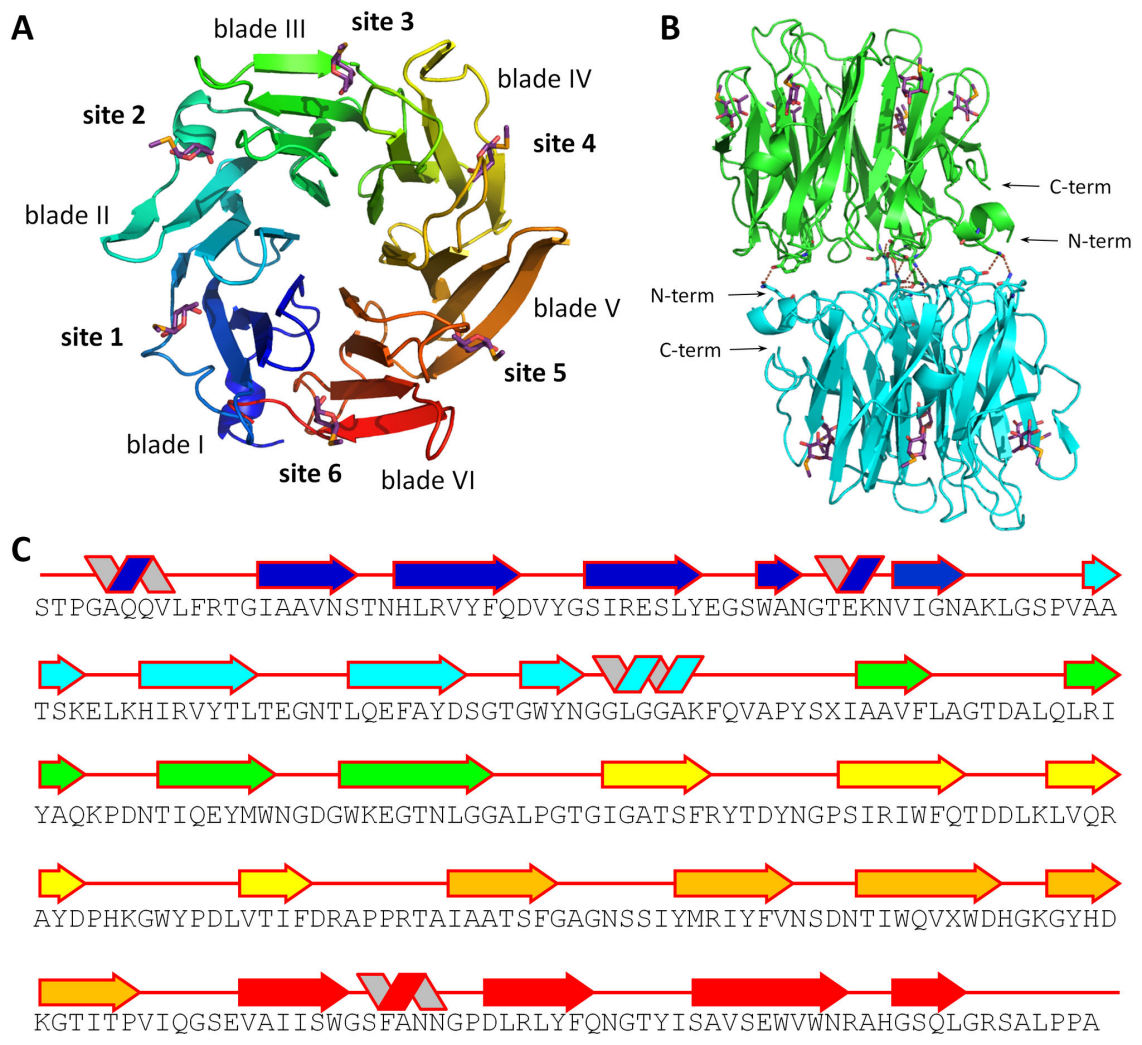
3D structure of AFL complexed with MeSeFuc. AFL monomer (chain A) overall architecture with individual blades and binding sites labelled (A) and side-view of AFL dimer (chains A and B) with intermonomer contacts shown (B). MeSeFuc ligands are shown as purple sticks. AFL protein sequence with secondary structure elements highlighted in colors according to panel A (C).

AFL crystallizes as a dimer that is formed by two monomers in pseudo 2-fold axial symmetry (Figure 4). The monomers directly interact using two loops in the region of blade V – one between the second and the third β-sheet and the other connecting the fourth β-sheet with the next β-blade. The side chains of Asn238 and Gln262 and backbone of Asn235, Ser236 and Gly263 interact with their counterparts from the other monomer. Additional hydrogen bonds are formed by the Gln7, Tyr109 and Asn134 of each chain. There are no stacking interactions between the two monomers. Other polar contacts between monomers are mediated by water molecules, while hydrophobic interactions only make a marginal contribution to oligomerization. In contrast to the AAL dimer, where only the loops of blades 6, 1 and 2 come into contact upon dimerization and the N- and C- termini are crucial [16], the AFL dimer interface is formed by the loops of all six blades and only the N-terminus is involved, not the C-terminus.

### Binding sites

Electron density corresponding to MeSeFuc revealed six binding sites per monomer. They are located on the opposite side of the molecule to the N- and C- termini, each of them between two adjacent blades (Figure 4). According to the nomenclature used previously for the AAL structure [16], site 1 is located between blades I and II and so on, finishing with site 6 between blade VI and blade I.

**Figure 5:**
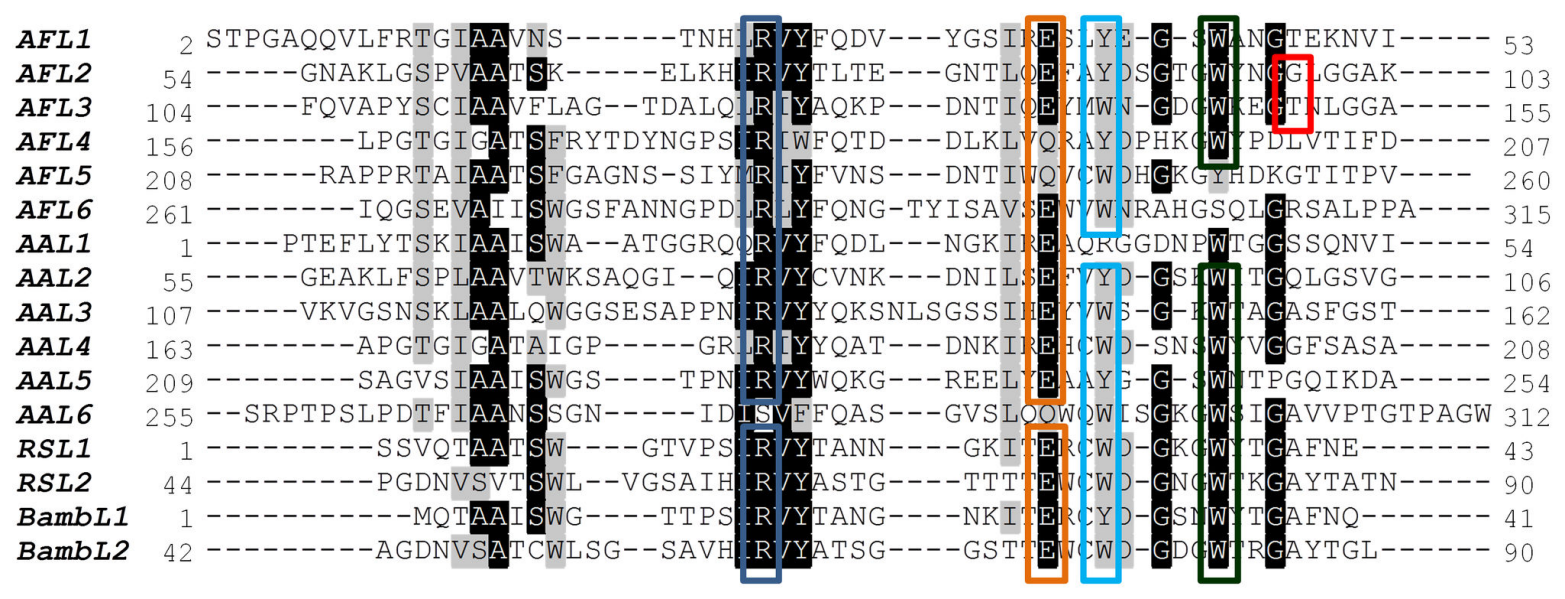
Multiple sequence alignment of AFL, AAL and RSL repetitions based on crystal structures. The corresponding PDB-deposited structures 4agi, 1ofz and 2bt9 were analysed. Boxed residues are involved in Fuc binding via a stacking interaction (cyan Trp/Tyr) or hydrogen bonds (blue Arg, orange Glu/Gln, green Trp and red non-conserved residues).

**Figure 6:**
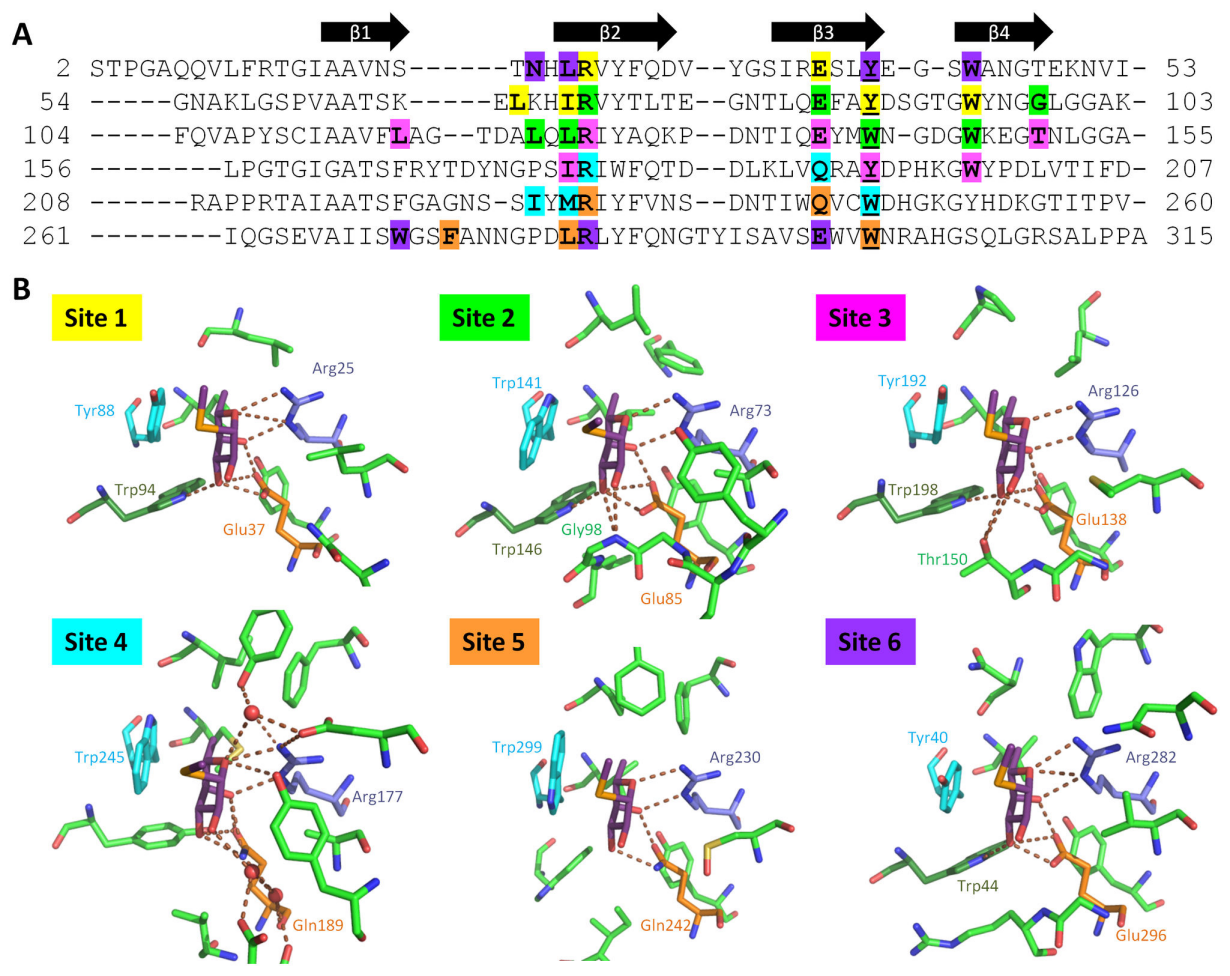
AFL binding sites. (A) The sequence of AFL six repetitions with indication of main secondary structure elements. Amino acids forming binding sites are highlighted by color corresponding to panel B. (B) The six AFL binding sites of chain A with MeSeFuc ligand. Colour code: Magenta – MeSeFuc, cyan – stacking residue Tyr/Trp, dark blue – conserved Arg, orange – conserved Glu/Gln, dark green – H-bond-forming Trp, light green – other residues in ligand proximity, water molecules involved in sugar binding shown as red spheres, hydrogen bonds formed by ligand shown as brown dashed lines.

Binding sites are pocket-shaped, composed of different sets of amino acids, although some of them are conserved or semi-conserved (Figure 5). Conserved Arg and Glu/Gln residues form one side of each binding site, establishing polar contacts with the O-3, O-4 and O-5 of bound Fuc. The opposing part of each site is formed by a Trp/Tyr residue that enables hydrophobic interaction with the apolar surface of Fuc (C-3, C-4 and C-5). Additional hydrogen bonds are observed between the saccharide and other Trp, Thr or Gly residues in binding sites 1, 2, 3 and 6. Binding site 4 includes one water bridge between the endocyclic oxygen O-5 of Fuc and Tyr168, Arg177 and Asp193 (Figure 6). Additional weak interactions are mainly between the C-6 of Fuc and non-polar amino acids in the binding site, including a semi-conserved Leu/Ile/Met in the pocket at the bottom of the site.

In the AFL/MeSeFuc complex, there is one additional MeSeFuc molecule located in proximity to site 2 of one monomer (A) and site 5 of a symmetrical monomer (B) in the neighboring crystal unit. The ligand is stabilized by hydrogen bonds connecting the ligand to backbone O-Asn96(A) and to OH-Tyr228(B) and backbone O-His247(B). A water bridge is formed with Gly98(A), and the Se-Se interaction between this molecule and MeSeFuc in site 2 (A). It is likely that this ligand is only coordinated due to the crystal packing.

### Immunostaining

**Figure 7:**
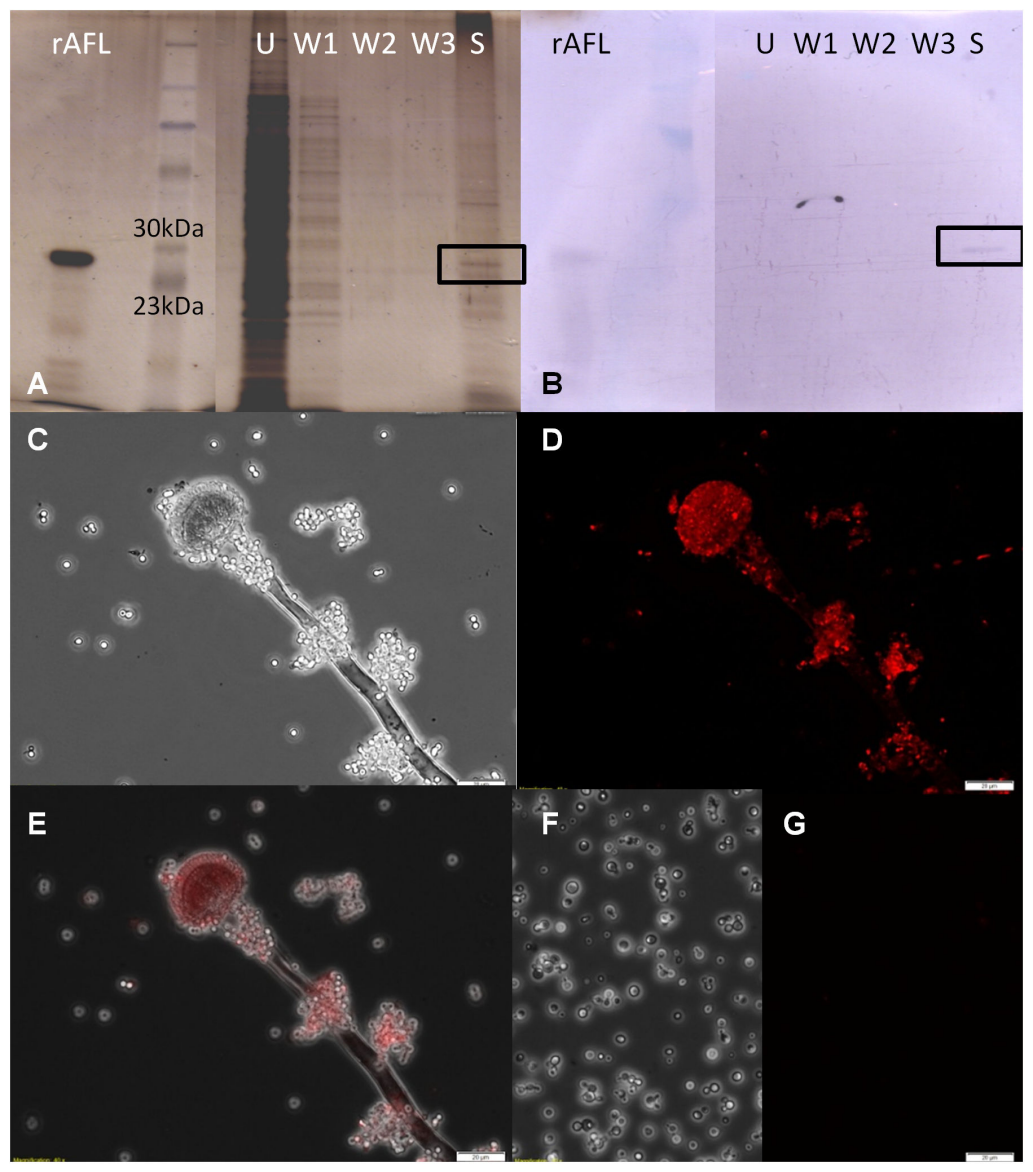
Immunostaining of *A. fumigatus* by anti-AFL polyclonal IgG. (A) *A. fumigatus* conidia lysate was loaded onto fucose-modified agarose resin and unbound proteins (line U), weakly bound proteins (W1-W3) and strongly interacting proteins (S) were analyzed on 12% SDS-PAGE with silver staining. Recombinant AFL (rAFL) was used for comparison. (B) Immunoblotting of gel A stained by polyclonal anti-AFL antibodies. Only recombinant AFL and one band of strongly interacting proteins identified as AFL (boxed) were stained. (C-G) Cultures from solid medium were harvested and immunolabeled. Images of conidiophores and several conidia clusters in visible light (C), fluorescence in the presence of anti-AFL polyclonal antibodies with Cy3-conjugated goat anti-rabbit IgG as secondary antibody (D) and a superposition of the two images (E). *P. pastoris* cells treated in the same way were used as a negative control - in visible light (F) and fluorescence (G).

The solved structure of AFL was used to rationally design suitable peptides for antibody production. A set of surface-exposed peptides was synthesized and used to generate anti-AFL rabbit polyclonal antibodies. The antibodies were used to detect the presence of AFL on the conidia and hyphae of *A. fumigatus*. Using immunoblotting, we could detect recombinant AFL (Figure S5). One protein band of the correct size was observed (Figure 7A-B), when analyzing the proteins from conidia lysate interacting with a resin with immobilized fucose. Subsequent MS analysis clearly identified this protein as AFL (data not shown). The observation of stained conidiophores (Figure 7C-E) shows the antibody binding, predominantly on the conidia, whereas the hyphal part exhibits no or very little interaction. *Pichia pastoris* GS 115 cells used as a negative control (Figure 7F-G), as well as rabbit IgG isotype control antibody (data not shown), did not result in a visible fluorescence signal. However, interaction of anti-AFL polyclonal antibodies was not inhibited by the synthetic peptide used for antibodies generation (data not shown). We also tested for the presence of fucose-binding epitopes on the surface of *A.fumigatus*. Even though it is only indirect evidence, we observed clear binding of the biotin-conjugated fucose-bearing polyacrylamide to the *Aspergillus*’ surface, while the streptavidin-conjugated fluorophore showed only marginal non-specific binding (Figure S6).

### Inflammatory effect of AFL

**Figure 8:**
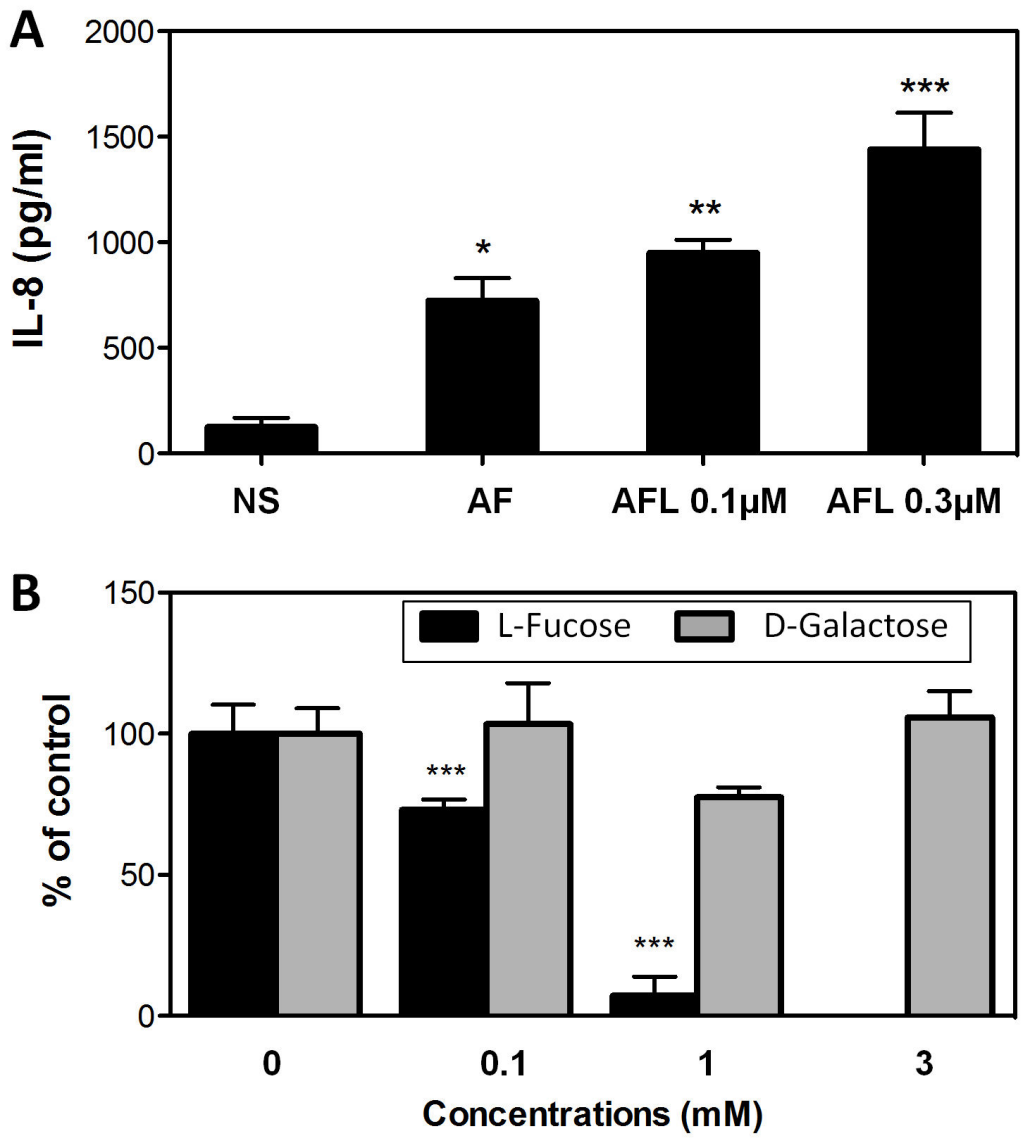
Activation of respiratory epithelial cells by AFL. Cells cultured in 24-well plates were incubated with AFL. (A) AFL was used at two different concentrations. Not stimulated cells (NS) and cells exposed to 10^6^ live conidia (AF) were used as negative and positive control, respectively. (B) AFL (0.3 µM) was preincubated for 1 hour with three different concentrations of either l-Fucose or d-Galactose prior addition to the cells. After 15 hour incubation, supernatants were collected and IL-8 concentrations were measured by ELISA. Each histogram is the mean ± S.E. of three experiments performed in triplicate. *, p < 0.05; ** p < 0.01; *** p < 0.001, significantly different from NS (Panel A) or from 0 (Panel B).

It has been shown that *A. fumigatus* can stimulate the immune system in different ways [35]. To test the influence of the AFL protein, human respiratory epithelial cells were exposed to the lectin and their IL-8 production was measured. The eliciting of IL-8 upon exposure to AFL was observed in a dose-dependent manner and was similar to the effect of exposure to *A. fumigatus* conidia (Figure 8A). For comparison, two other lectins of bacterial origin were tested as positive and negative controls. The amount of IL-8 produced was 702.7 ± 105.2 (mean ± S.E.; n=4; p<0.001 compared to resting cells) for treatment with 0.1 µM Bambl (fucose-specific lectin from *Burkholderia ambifaria* [20]) and 128.6 ± 21.4 (mean ± S.E.; n=4; ns compared to resting cells) with 0.5 µM BC2L-A (mannose-specific lectin from *Burkholderia cepacia complex* [36]), respectively. The effect of sugar recognition by AFL was examined by addition of various concentrations of l-fucose and d-galactose, respectively, both to stimulated and non-stimulated cells. The addition of 1 mM l-Fuc almost completely inhibited IL-8 production, whereas d-galactose did not significantly affect the stimulation of the epithelial cells by AFL (Figure 8B).

## Discussion

*Aspergillus fumigatus* is one of the most frequently detected opportunistic fungal pathogens. There are several potential lectins identified in its genome, but little is known about their properties and their role in infection and/or pathogenicity. The aim of this study was to describe the structure and basic binding properties of the lectin AFL from *A. fumigatus* and to determine its natural occurrence and possible role in pathogenicity.

Previous studies of *A. fumigatus* focused mainly on its carbohydrate moieties. The first reported case of a lectin from *A. fumigatus* being purified and partially characterized was in 2002 [11]. Although the size of the sialic acid binding protein examined by Tronchin et al. is in the same range as AFL, the serious difference in binding properties suggests that these are two distinct lectins from *A. fumigatus*. Recently, the existence of AFL was proven *in vivo*, however the structure and sugar-binding properties of the lectin were not described in detail [22].

AFL has strong sequence and structure similarities to the well-known AAL lectin from *Aleuria aurantia*, however we found differences, especially in their dimer formation and binding site organization. While five binding sites have been identified in AAL, AFL possesses six binding sites. Six binding sites were previously found in the β-propeller of two other structural homologues – RSL from *Ralstonia solanacearum* [19] and BambL from *Burkholderia ambifaria* [20]. However in these bacterial lectins, the β-propeller is formed by a trimerisation of shorter peptides, with two slightly different binding sites per peptide. The binding sites of AFL all differ in their structure, although the basic binding motif remains the same. Therefore AFL appears to be an ideal subject for the analysis of binding site variability.

The hemagglutination assay revealed binding preferences towards Fuc and its derivatives. This specificity has been detected for all members of the AAL lectin family. The lower affinities for the binding of AFL to l-galactose and the very low binding affinities to d-fructose and d-mannose that were observed correspond to the differences in the structures of these saccharides. The submillimolar levels of observed MICs are rather high compared to the K_d_’s of AFL homologues, but as with AAL, the presence of binding sites with different affinities is highly probable. The glycan array data revealed the ability of AFL to bind to various fucosylated oligosaccharides. Surprisingly, most of the oligosaccharides are less tightly bound than the immobilized fucose monosaccharide. Fucosylated disaccharides and some small oligosaccharides are however also strongly bound by AFL. Of the blood group determinants, Le^Y^ is the most dominantly bound epitope of all, thus AFL could be categorized as a Le^Y^-preferring lectin. However, the direct link between the epitope and protein structure can't be easily set. The structure analysis of homologous BambL protein with H type trisaccharide revealed that although there are only two different binding sites in BambL, oligosaccharide adopts different conformation in each of them [20]. The six binding sites of AFL are all surface exposed, so the additional stabilizing contacts between the lectin and Le^Y^ epitopes may be created through various amino acids. 

A general tendency of AFL lectin to prefer epitopes bound through longer linkers was observed, as well as a low affinity towards branched saccharides. This demonstrates the importance of steric hindrance that affects the ligand binding. The biologically important core α1-6 Fuc is also recognized, although the binding is generally weaker than with terminal epitopes. This is promising in terms of the future application of AFL as a core-Fuc detecting agent to join the previously described lectins, such as AAL from *Aleuria aurantia* and PhosL from *Pholiota squarrosa* [37] that posses the ability to recognize α1-6 Fuc.

We performed our study on the recombinant protein, demonstrating its production in *E.coli* cells with a retained activity and structure. The input *afl* gene was gained from an *A. fumigatus* cDNA library, so the gene is transcribed *in vivo*. The AFL-specific antibody staining of conidia lysates revealed a production of active AFL at the protein level. AFL (named FleA by analogy to the *A. oryzae* lectin) was also recently identified in the secreted proteome of *A. fumigatus* [38] and isolated from its mycelial mass [22]. No targeting signal is predicted, however other lectins without a defined secretion pathway have already been determined to be present on the cell surface [39,40]. The exposure of the protein to the cell environment has several consequences. One of the main functions of lectins is the attachment of cells to various surfaces, including host epithelia [41]. The binding properties of lectins determine the preferred host and may be the factor that distinguishes between harmless and pathogenic microorganisms. AFL specifically recognizes fucose with high affinity, and this carbohydrate is widely found on human cells [42]. We believe that AFL may be present on the conidial surface and it could be the protein responsible for conidia attachment to the human lung epithelium, which is the first step in *Aspergillus* infection [3].

The connection between inflammation and fucose-binding lectins has recently been described for another lung pathogen, *B. cenocepacia*. The observed production of IL-8 upon AFL treatment of epithelial cells was comparable to the effect caused by the lectin BC2L-C that shows structural similarities with Tumor Necrosis Factor (TNF) [40], and it can be specifically inhibited by l-fucose in a dose dependent manner. As such, AFL could be one of the factors responsible for the pro-inflammatory effect of *A. fumigatus* [35,43]. In addition, AFL was reported to be recognized by human antibodies isolated from the sera of allergic bronchopulmonary aspergillosis patients [38], which makes it a candidate as an important virulence factor.

In conclusion, our study describes a novel fucose-specific lectin, AFL from *A. fumigatus*, which prefers the Le^Y^ epitope, with the ability to recognize core α1-6 Fuc. The variable composition of its binding sites enables it to bind to various fucosylated saccharides, including those expressed on human epithelia. The probable presence of AFL on the conidia and its pro-inflammatory effect makes it likely to play a role in the *A. fumigatus* colonization of human bodies and development of the infection.

## Supporting Information

Figure S1 **Phylogram of fungal homologues of AAL lectin.** Homologous sequences were identified using NCBI-Blast algorithm and phylogeny distances computed by ClustalW2.0. AFL is more related to most of other fungal homologues than to AAL (AFL and AAL shown in bold).
(TIF)

Figure S2 **Multiple sequence alignment of AFL-coding genes.** Af293 ˗ partial mRNA of *fleA* gene gi: 70996856, Af293_complete ˗ part of Af293 whole genome sequence gb: AAHF01000003.1, Af_cDNA ˗ sequence of PCR product with commercial cDNA library as a template, Af210_complete ˗ part of Af210 whole genome sequence gb: AFXM01000425.1. Regions identified as introns are shaded grey, mutations are shaded yellow. Different stop codon in Af_cDNA was introduced during PCR.
(TIF)

Figure S3 **AFL expression and purification.** Analysis on 15% SDS-PAGE. Cell culture before induction (1) and after 3 hr expression (2), insoluble (3) and soluble (4) fraction of cell lysate. Fractions eluted from mannose-agarose column (5-7). Bands of app 34 kDa containing AFL are boxed.
(TIF)

Figure S4 ***Schematical representation* of oligosaccharides used in glycanarray analysis.** Schemes use convention according to Taylor and Drickamer [44]. Numbers correspond to the bars in Figure 1, numbers in brackets correspond to numbers of glycan in glycan array chip (listed in Table S1).
(TIF)

Figure S5 **AFL immunoblot staining.** Two-fold serial dilutions of recombinant AFL starting at 250ng were made in 1x Novex NuPAGE LDS sample buffer with reducing agent (Life Technologies, Grand Island, NY) and incubated at 70°C for 10 minutes prior to running on a Novex NuPAGE 4-12% SDS PAGE gel (Life Technologies, Grand Island, NY). After transfer to a nitrocellulose membrane, the immunoblot was blocked overnight at 4°C with 5% milk in TBS + 0.1% Tween and stained with anti-AFL polyclonal antibody at 1μg/ml for 2 hours at room temperature. After washing, positive reactivity was detected using a donkey anti-rabbit IgG-HRP conjugated secondary antibody at a 1:10000 dilution (Jackson Immuno, Westgrove, PA) and ECL plus (GE Healthcare, Pittsburg, PA) prior to imaging on film.
(TIF)

Figure S6 ***A. fumigatus* interaction with fucose-polyacrylamide-biotin conjugate.** *A. fumigatus* sample was incubated with fucose-polyacrylamide-biotin conjugate and subsequently with AlexaFluor488-streptavidin conjugate for visualisation (see Material and Methods). The preparate was observed in visible light (A), the green fluorescence (B) and both images merged (C). Upon omitting the fucose-modified conjugate, only maginal non-specific binding of streptavidin conjugate was observed - visible light (D), green fluorescence (E) and both images merged (F).
(TIF)

Table S1 **Glycan array (v4.1) data for AFL arranged by the averaged relative binding calculated from three concentrations.** Avg is the average value from 10 measurements, %CV is standard deviation in %, rank is relative binding in % compared to best recognized oligosaccharide for particular concentration and avg rank is the average of ranks.
(XLSX)

Table S2 **Oligosaccharide structures corresponding to Figure 2.**
(DOCX)

Table S3 **Data collection and phasing statistics for AFL structure.**
(DOCX)
