# Supplementary figures and images for: A Soluble Fucose-Specific Lectin from Aspergillus fumigatus Conidia - Structure, Specificity and Possible Role in Fungal Pathogenicity
Source: PLoS One. 2013 Dec 10;8(12):e83077. doi: 10.1371/journal.pone.0083077 (PMC3858362; doi:10.1371/journal.pone.0083077)

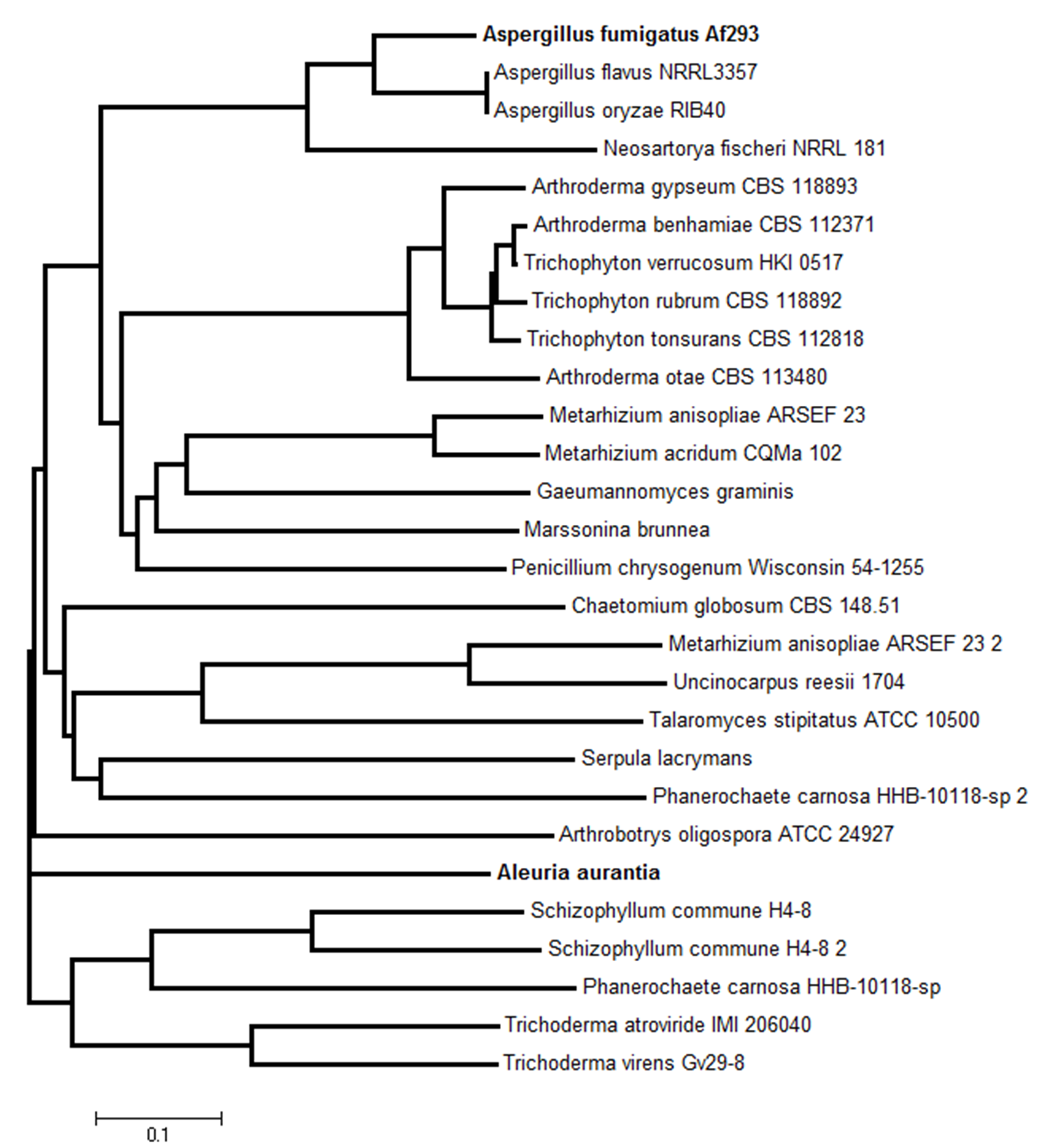

Supplement: Figure S1 — Phylogram of fungal homologues of AAL lectin. Homologous sequences were identified using NCBI-Blast algorithm and phylogeny distances computed by ClustalW2.0. AFL is more related to most of other fungal homologues than to AAL (AFL and AAL shown in bold). (TIF) [file pone.0083077.s001.tif]

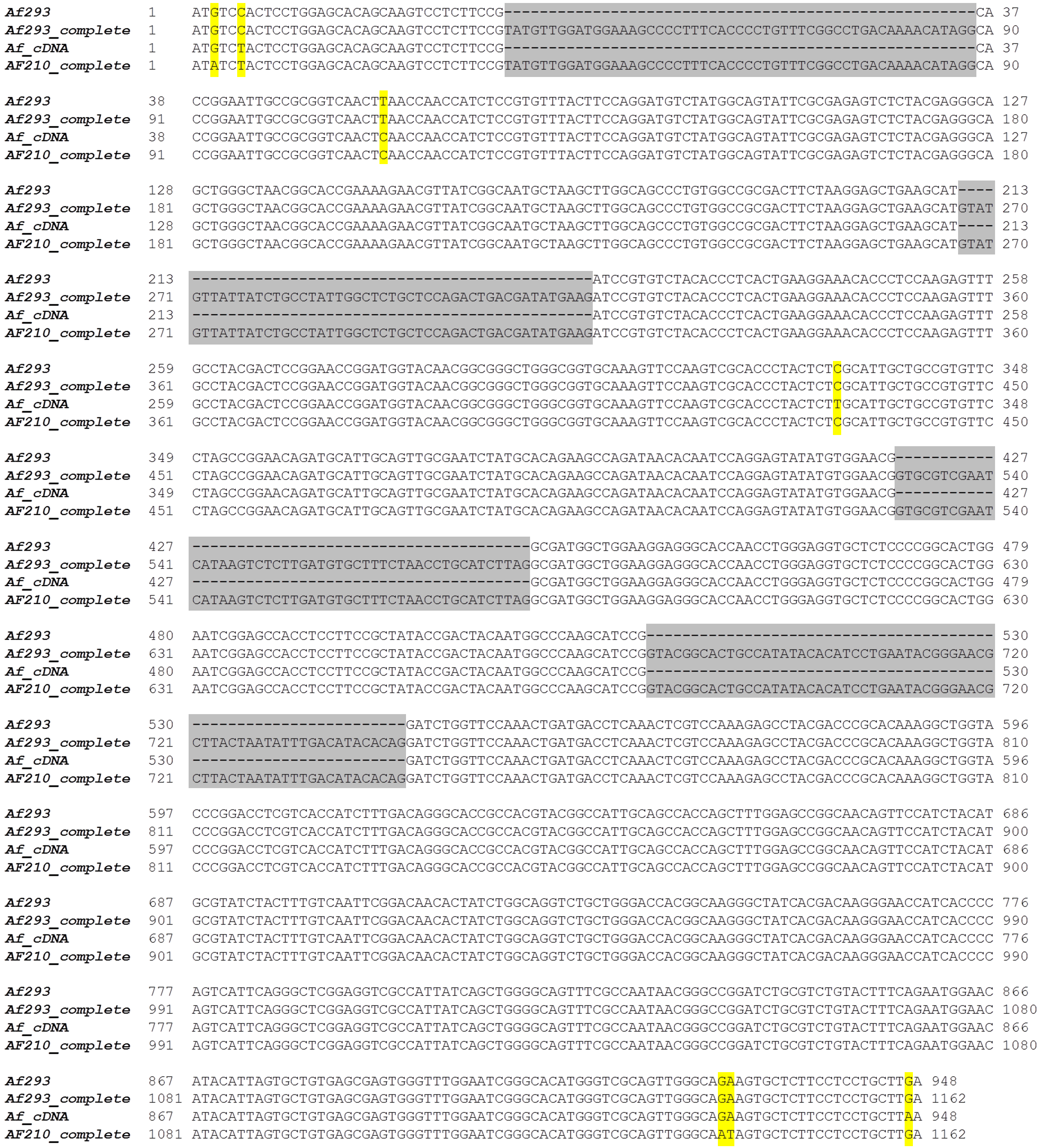

Supplement: Figure S2 — Multiple sequence alignment of AFL-coding genes. Af293 ˗ partial mRNA of fleA gene gi: 70996856, Af293_complete ˗ part of Af293 whole genome sequence gb: AAHF01000003.1, Af_cDNA ˗ sequence of PCR product with commercial cDNA library as a template, Af210_complete ˗ part of Af210 whole genome sequence gb: AFXM01000425.1. Regions identified as introns are shaded grey, mutations are shaded yellow. Different stop codon in Af_cDNA was introduced during PCR. (TIF) [file pone.0083077.s002.tif]

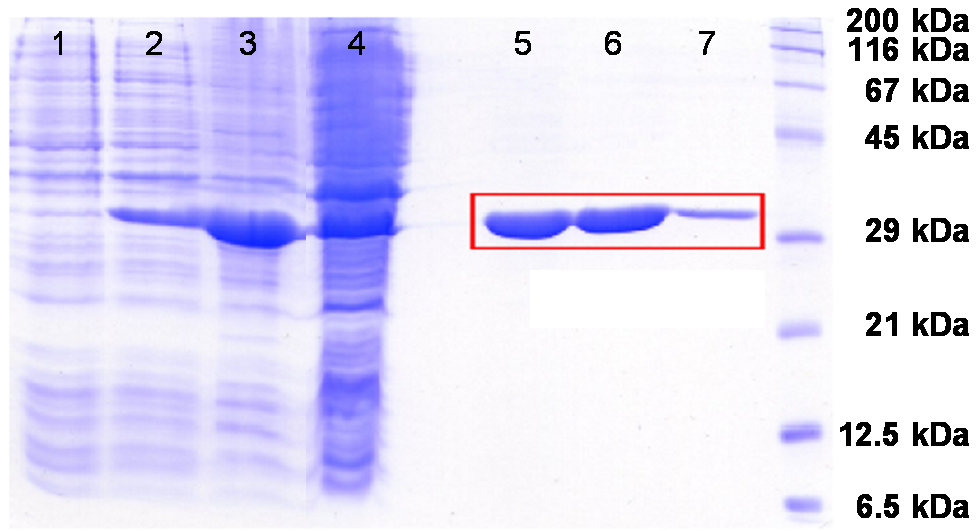

Supplement: Figure S3 — AFL expression and purification. Analysis on 15% SDS-PAGE. Cell culture before induction (1) and after 3 hr expression (2), insoluble (3) and soluble (4) fraction of cell lysate. Fractions eluted from mannose-agarose column (5-7). Bands of app 34 kDa containing AFL are boxed. (TIF) [file pone.0083077.s003.tif]

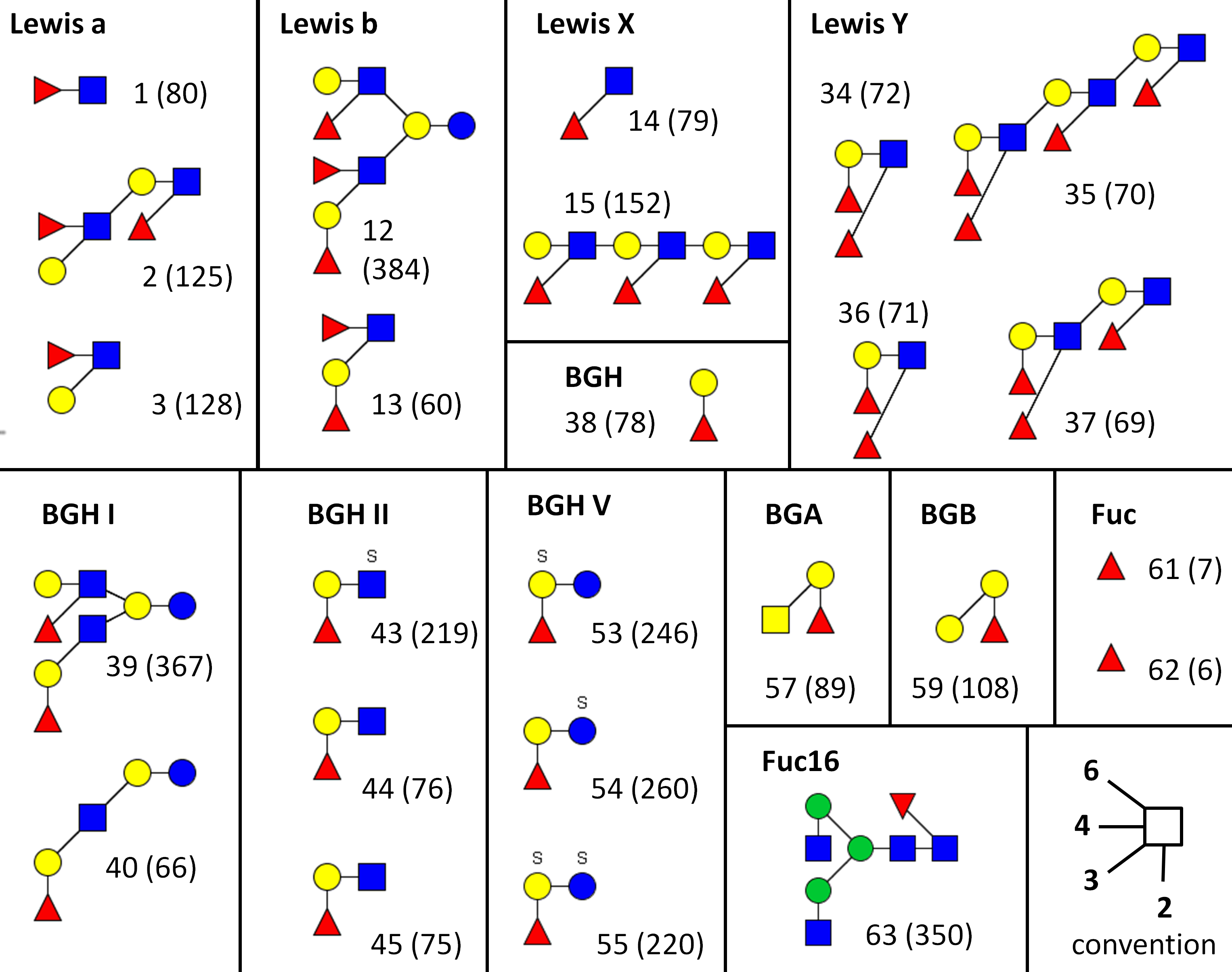

Supplement: Figure S4 — Schematical representation of oligosaccharides used in glycanarray analysis. Schemes use convention according to Taylor and Drickamer [44]. Numbers correspond to the bars in Figure 1, numbers in brackets correspond to numbers of glycan in glycan array chip (listed in Table S1). (TIF) [file pone.0083077.s004.tif]

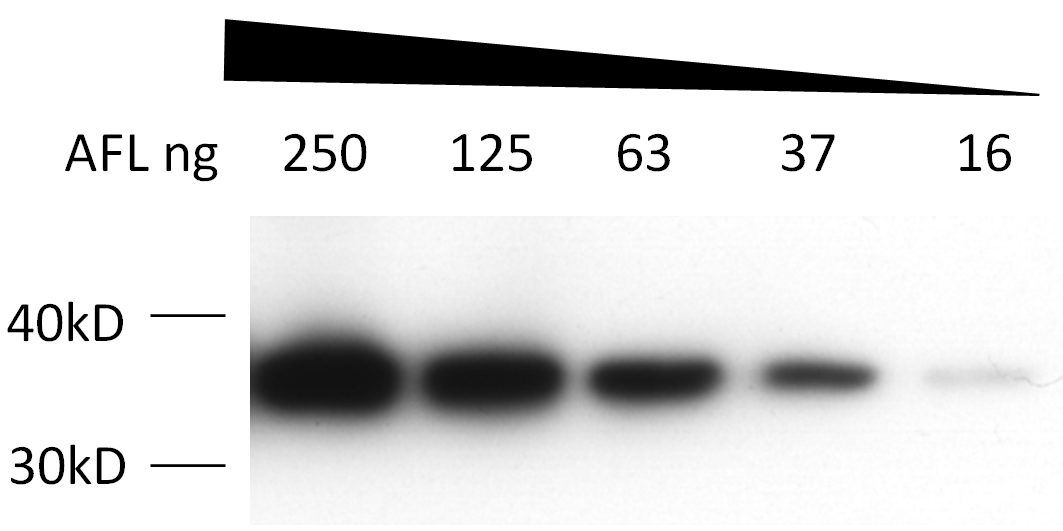

Supplement: Figure S5 — AFL immunoblot staining. Two-fold serial dilutions of recombinant AFL starting at 250ng were made in 1x Novex NuPAGE LDS sample buffer with reducing agent (Life Technologies, Grand Island, NY) and incubated at 70°C for 10 minutes prior to running on a Novex NuPAGE 4-12% SDS PAGE gel (Life Technologies, Grand Island, NY). After transfer to a nitrocellulose membrane, the immunoblot was blocked overnight at 4°C with 5% milk in TBS + 0.1% Tween and stained with anti-AFL polyclonal antibody at 1μg/ml for 2 hours at room temperature. After washing, positive reactivity was detected using a donkey anti-rabbit IgG-HRP conjugated secondary antibody at a 1:10000 dilution (Jackson Immuno, Westgrove, PA) and ECL plus (GE Healthcare, Pittsburg, PA) prior to imaging on film. (TIF) [file pone.0083077.s005.tif]

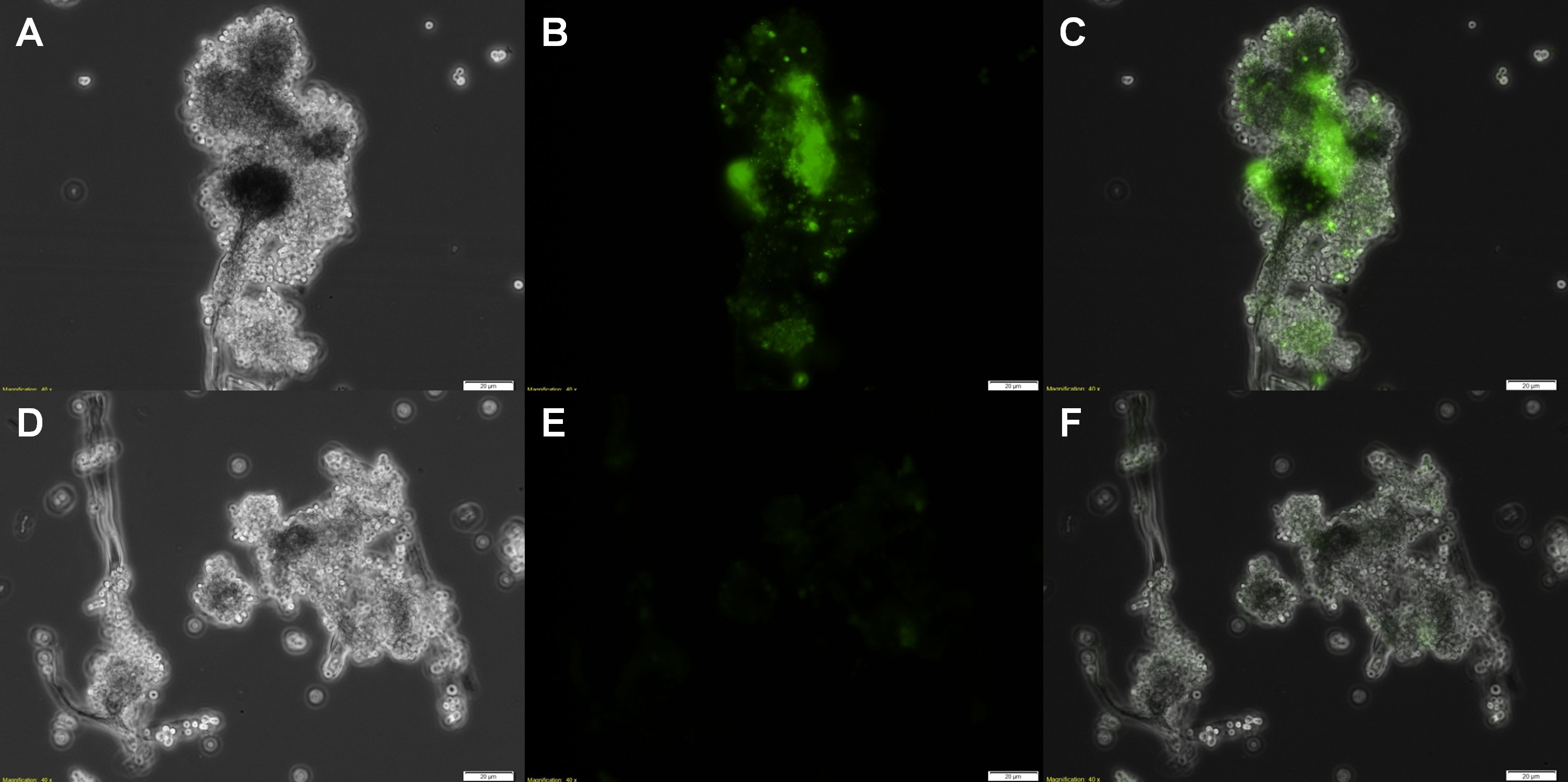

Supplement: Figure S6 — A. fumigatus interaction with fucose-polyacrylamide-biotin conjugate. A. fumigatus sample was incubated with fucose-polyacrylamide-biotin conjugate and subsequently with AlexaFluor488-streptavidin conjugate for visualisation (see Material and Methods). The preparate was observed in visible light (A), the green fluorescence (B) and both images merged (C). Upon omitting the fucose-modified conjugate, only maginal non-specific binding of streptavidin conjugate was observed - visible light (D), green fluorescence (E) and both images merged (F). (TIF) [file pone.0083077.s006.tif]
