# Supplementary material for: A Soluble Fucose-Specific Lectin from Aspergillus fumigatus Conidia - Structure, Specificity and Possible Role in Fungal Pathogenicity
Source: PLoS One. 2013 Dec 10;8(12):e83077. doi: 10.1371/journal.pone.0083077 (PMC3858362; doi:10.1371/journal.pone.0083077)
Supplement: Table S2 — Oligosaccharide structures corresponding to Figure 2. (DOCX) [file pone.0083077.s008.docx]

Table S2: Oligosaccharide structures corresponding to Fig. 2.

| **Group** | **Bar** | **v4.1 Chip Number** | **Glycan Structure** |
| --- | --- | --- | --- |
| Lea | 1 | 80 | Fucα1-4GlcNAcβ-Sp8 |
|  | 2 | 125 | Galβ1-3(Fucα1-4)GlcNAcβ1-3Galβ1-4(Fucα1-3)GlcNAcβ-Sp0 |
|  | 3 | 128 | Galβ1-3(Fucα1-4)GlcNAcβ-Sp8 |
|  | 4 | 127 | Galβ1-3(Fucα1-4)GlcNAcβ-Sp0 |
|  | 5 | 27 | [3OSO3]Galβ1-3(Fucα1-4)GlcNAcβ-Sp8 |
|  | 6 | 129 | Galβ1-3(Fucα1-4)GlcNAc-Sp8 |
|  | 7 | 275 | Galβ1-3(Fucα1-4)GlcNAcβ1-3Galβ1-3(Fucα1-4)GlcNAcβ-Sp0 |
|  | 8 | 237 | Neu5Acα2-3Galβ1-3(Fucα1-4)GlcNAcβ-Sp8 |
|  | 9 | 238 | Neu5Acα2-3Galβ1-3(Fucα1-4)GlcNAcβ1-3Galβ1-4(Fucα1-3)GlcNAcβ-Sp0 |
|  | 10 | 329 | Neu5Acα2-3Galβ1-3(Fucα1-4)GlcNAcβ1-3Galβ1-3(Fucα1-4)GlcNAcβ-Sp0 |
|  | 11 | 126 | Galβ1-3(Fucα1-4)GlcNAcβ1-3Galβ1-4GlcNAcβ-Sp0 |
| Leb + LeX | 12 | 384 | Fucα1-2Galβ1-3(Fucα1-4)GlcNAcβ1-3(Galβ1-4(Fucα1-3)GlcNAcβ1-6)Galβ1-4Glc-Sp21 |
| Leb | 13 | 60 | Fucα1-2Galβ1-3(Fucα1-4)GlcNAcβ-Sp8 |
| LeX | 14 | 79 | Fucα1-3GlcNAcβ-Sp8 |
|  | 15 | 152 | Galβ1-4(Fucα1-3)GlcNAcβ1-4Galβ1-4(Fucα1-3)GlcNAcβ1-4Galβ1-4(Fucα1-3)GlcNAcβ-Sp0 |
|  | 16 | 150 | Galβ1-4(Fucα1-3)GlcNAcβ-Sp8 |
|  | 17 | 251 | Neu5Acα2-3Galβ1-4(Fucα1-3)GlcNAcβ1-3Galβ1-4(Fucα1-3)GlcNAcβ1-3Galβ1-4(Fucα1-3)GlcNAcβ-Sp0 |
|  | 18 | 290 | Galβ1-4(Fucα1-3)GlcNAcβ1-3Galβ1-3(Fucα1-4)GlcNAcβ-Sp0 |
|  | 19 | 151 | Galβ1-4(Fucα1-3)GlcNAcβ1-4Galβ1-4(Fucα1-3)GlcNAcβ-Sp0 |
|  | 20 | 250 | Neu5Acα2-3Galβ1-4(Fucα1-3)[6OSO3]GlcNAcβ-Sp8 |
|  | 21 | 325 | Fucα1-3(Galβ1-4)GlcNAcβ1-2Manα1-3(Fucα1-3(Galβ1-4)GlcNAcβ1-2Manα1-6)Manβ1-4GlcNAcβ1-4GlcNAcβ-Sp20 |
|  | 22 | 288 | Galβ1-4(Fucα1-3)[6OSO3]GlcNAc-Sp0 |
|  | 23 | 254 | Neu5Acα2-3Galβ1-4(Fucα1-3)GlcNAcβ1-3Galβ-Sp8 |
|  | 24 | 31 | [3OSO3]Galβ1-4(Fucα1-3)GlcNAc-Sp0 |
|  | 25 | 228 | Neu5Acα2-3(6-O-Su)Galβ1-4(Fucα1-3)GlcNAcβ-Sp8 |
|  | 26 | 32 | [3OSO3]Galβ1-4(Fucα1-3)GlcNAcβ-Sp8 |
|  | 27 | 255 | Neu5Acα2-3Galβ1-4(Fucα1-3)GlcNAcβ1-3Galβ1-4GlcNAcβ-Sp8 |
|  | 28 | 217 | [3OSO3]Galβ1-4(Fucα1-3)[6OSO3]GlcNAc-Sp8 |
|  | 29 | 149 | Galβ1-4(Fucα1-3)GlcNAcβ-Sp0 |
|  | 30 | 340 | GlcNAcα1-4Galβ1-4GlcNAcβ1-3Galβ1-4(Fucα1-3)GlcNAcβ1-3Galβ1-4(Fucα1-3)GlcNAcβ-Sp0 |
|  | 31 | 253 | Neu5Acα2-3Galβ1-4(Fucα1-3)GlcNAcβ-Sp8 |
|  | 32 | 382 | Galβ1-3GlcNAcβ1-3(Galβ1-4(Fucα1-3)GlcNAcβ1-6)Galβ1-4Glc-Sp21 |
|  | 33 | 84 | [3OSO3]Galβ1-4(Fucα1-3)Glc-Sp0 |
| LeY | 34 | 72 | Fucα1-2Galβ1-4(Fucα1-3)GlcNAcβ-Sp8 |
|  | 35 | 70 | Fucα1-2Galβ1-4(Fucα1-3)GlcNAcβ1-3Galβ1-4(Fucα1-3)GlcNAcβ1-3Galβ1-4(Fucα1-3)GlcNAcβ-Sp0 |
|  | 36 | 71 | Fucα1-2Galβ1-4(Fucα1-3)GlcNAcβ-Sp0 |
|  | 37 | 69 | Fucα1-2Galβ1-4(Fucα1-3)GlcNAcβ1-3Galβ1-4(Fucα1-3)GlcNAcβ-Sp0 |
| BG H | 38 | 78 | Fucα1-2Galβ-Sp8 |
| BG HI + LeX | 39 | 367 | Fucα1-2Galβ1-3GlcNAcβ1-3(Galβ1-4(Fucα1-3)GlcNAcβ1-6)Galβ1-4Glc-Sp21 |
| BG HI | 40 | 66 | Fucα1-2Galβ1-3GlcNAcβ1-3Galβ1-4Glcβ-Sp10 |
|  | 41 | 65 | Fucα1-2Galβ1-3GlcNAcβ1-3Galβ1-4Glcβ-Sp8 |
|  | 42 | 68 | Fucα1-2Galβ1-3GlcNAcβ-Sp8 |
| BG HII | 43 | 219 | Fucα1-2Galβ1-4[6OSO3]GlcNAc-Sp8 |
|  | 44 | 76 | Fucα1-2Galβ1-4GlcNAcβ-Sp8 |
|  | 45 | 75 | Fucα1-2Galβ1-4GlcNAcβ-Sp0 |
|  | 46 | 74 | Fucα1-2Galβ1-4GlcNAcβ1-3Galβ1-4GlcNAcβ1-3Galβ1-4GlcNAcβ-Sp0 |
|  | 47 | 73 | Fucα1-2Galβ1-4GlcNAcβ1-3Galβ1-4GlcNAcβ-Sp0 |
|  | 48 | 451 | Fucα1-2Galβ1-4GlcNAcβ1-3(Fucα1-2Galβ1-4GlcNAcβ1-6)GalNAc-Sp14 |
|  | 49 | 360 | Fucα1-2Galβ1-4GlcNAcβ1-2Manα1-3(Fucα1-2Galβ1-4GlcNAcβ1-2Manα1-6)Manβ1-4GlcNAcβ1-4GlcNAcβ-Sp20 |
|  | 50 | 218 | Fucα1-2[6OSO3]Galβ1-4GlcNAc-Sp0 |
|  | 51 | 419 | Fucα1-2Galβ1-4GlcNAcβ1-2Manα1-3(Fucα1-2Galβ1-4GlcNAcβ1-2Manα1-6)Manβ1-4GlcNAcβ1-4(Fucα1-6)GlcNAcβ-Sp22 |
|  | 52 | 445 | Fucα1-2Galβ1-4GlcNAcβ1-2(Fucα1-2Galβ1-4GlcNAcβ1-4)Manα1-3(Fucα1-2Galβ1-4 GlcNAcβ1-2Manα1-6)Manβ1-4GlcNAcβ1-4GlcNAcβ-Sp12 |
| BG HV | 53 | 246 | Fucα1-2[6OSO3]Galβ1-4Glc-Sp0 |
|  | 54 | 260 | Fucα1-2Galβ1-4[6OSO3]Glc-Sp0 |
|  | 55 | 220 | Fucα1-2[6OSO3]Galβ1-4[6OSO3]Glc-Sp0 |
|  | 56 | 77 | Fucα1-2Galβ1-4Glcβ-Sp0 |
| BG A | 57 | 89 | GalNAcα1-3(Fucα1-2)Galβ-Sp8 |
|  | 58 | 90 | GalNAcα1-3(Fucα1-2)Galβ-Sp18 |
| BG B | 59 | 108 | Galα1-3(Fucα1-2)Galβ-Sp18 |
|  | 60 | 107 | Galα1-3(Fucα1-2)Galβ-Sp8 |
| Fuc | 61 | 7 | Fucα-Sp9 |
|  | 62 | 6 | Fucα-Sp8 |
| Fuc16 | 63 | 350 | GlcNAcβ1-2Manα1-3(GlcNAcβ1-2Manα1-6)Manβ1-4GlcNAcβ1-4(Fucα1-6)GlcNAcβ-Sp22 |
|  | 64 | 351 | Galβ1-4GlcNAcβ1-2Manα1-3(Galβ1-4GlcNAcβ1-2Manα1-6)Manβ1-4GlcNAcβ1-4(Fucα1-6)GlcNAcβ-Sp22 |
|  | 65 | 352 | Galβ1-3GlcNAcβ1-2Manα1-3(Galβ1-3GlcNAcβ1-2Manα1-6)Manβ1-4GlcNAcβ1-4(Fucα1-6)GlcNAcβ-Sp22 |

Structures are grouped according to terminal epitope. If more than one type of epitope is present, note is made in Group column. Bar stands for rank in the chart. Spacer abbreviations in chart taken from Consortium for Functional Glycomics: Sp0 – -CH_2_CH_2_NH_2_, Sp8 – -CH_2_CH_2_CH_2_NH_2_, Sp9 – -CH_2_CH_2_CH_2_CH_2_CH_2_NH_2_, Sp10 – -NHCOCH_2_NH, Sp14 – Threonine, Sp18 – -O(CH_2_)_3_NHCO(CH_2_)_5_NH_2_, Sp20 – GENR, Sp21 – -N(CH_3_)-O-(CH_2_)_2_-NH_2_, Sp22 – NST
