# Supplementary material for: A Soluble Fucose-Specific Lectin from Aspergillus fumigatus Conidia - Structure, Specificity and Possible Role in Fungal Pathogenicity
Source: PLoS One. 2013 Dec 10;8(12):e83077. doi: 10.1371/journal.pone.0083077 (PMC3858362; doi:10.1371/journal.pone.0083077)
Supplement: Table S3 — Data collection and phasing statistics for AFL structure. (DOCX) [file pone.0083077.s009.docx]

Table S3. Data collection and phasing statistics for AFL structure.

| ***Data collection*** |  |
| --- | --- |
| **Beamline** | ID14-4; ESRF |
| **Space group** | P1 |
| **Resolution (Å)** | 43.79 - 1.60 |
| **Highest resolution shell (Å)** | 1.69 - 1.60 |
| **Wavelength (Å)** | 0.980 |
| **Cell dimensions** |  |
| a (Å) | 46.69 |
| b (Å) | 79.85 |
| c (Å) | 84.39 |
| α (degrees) | 91.08 |
| β (degrees) | 88.89 |
| γ (degrees) | 103.00 |
| **Total number of observations** | 583984 |
| **Unique reflections** | 150144 (21406)* |
| **Average multiplicity** | 3.9 (3.9) |
| **Rmerge (%)** | 0.042 (0.273) |
| **Average I/σ(I)** | 19.4 (4.1) |
| **Completeness (%)** | 95.7 (93.4) |
| **Completeness for anomalous data (%)** | 91.8 (91.4) |
| **Wilson B-factor *(Å^2^)*** | 10.1 |
|  |  |
| ***Refinement statistics*** |  |
| **Amino acids** | 4 x 314 |
| **Protein atoms** | 9793 |
| **Solvent atoms** | 1161 |
| **Sugar atoms** | 312 |
| **Resolution limits (Å)** | 43.79-1.60 |
| **R factor (observations)** | 0.152 (142614) |
| **R free (observations)** | 0.184 (7525) |
| **RMSD bonds (Å)** | 0.016 |
| **RMSD Angles (degrees)** | 1.681 |
| **Cruickshank's DPI** | 0.082 |
| **Number of outliers on Ramachandran plot** | 1 |
| **Average B_iso_ (Å^2^)** |  |
| All atoms | 12.83 |
| Protein atoms | 11.86 |
| Solvent atoms | 20.79 |

Structure with MeSeFuc ligand (PDB code: 4agi). *values in parenthesis refers to the highest resolution shell
